# Supplementary material for: Synthesis, Characterisation, Biological Evaluation and In Silico Studies of Quinoline–1,2,3-Triazole–Anilines as Potential Antitubercular and Anti-HIV Agents
Source: Molecules. 2025 May 10;30(10):2119. doi: 10.3390/molecules30102119 (PMC12114169; doi:10.3390/molecules30102119)

SUPPLEMENTARY INFORMATION

Synthesis, Characterisation, Biological Evaluation and In Silico Studies of Quinoline-1,2,3-Triazole-Anilines as Potential Antitubercular and Anti-HIV Agents

Snethemba S. Magwaza[1], Darian Naidu[2], Oluwatoba E. Oyeneyin[1,3], Sibusiso Senzani[4], Nompumelelo P. Mkhwanazi[2], Matshawandile Tukulula[1]*

[1]School of Chemistry and Physics, University of KwaZulu Natal, Westville Campus, Durban 4000, South Africa; magwazas@ukzn.ac.za (S.S.M)

[2]HIV Pathogenesis Programme, Doris Duke Medical Research Institute, School of Laboratory Medicine and Medical Sciences, College of Heath Health Sciences, University of KwaZulu-Natal, Durban, 4001, South Africa; 218052764@stu.ukzn.ac.za (D.N.); mkhwanazi@ukzn.ac.za (N.P.M)

[3]Department of Chemical Sciences, Adekunle Ajasin University, Akungba-Akoko, 34311, Nigeria; Oluwatoba.oyeneyin@aaua.edu.ng (O.E.O)

[4]School of Laboratory Medicine and Medical Science, College of Heath Health Sciences, University of KwaZulu Natal, Medical School Campus, Durban 4001, South Africa; senzanis@ukzn.ac.za (S.S)

*Corresponding author(s): Email: tukululam@ukzn.ac.za Tel 031 260 8756 Fax 031 260 3091

Cell viability plot for the synthesised compounds

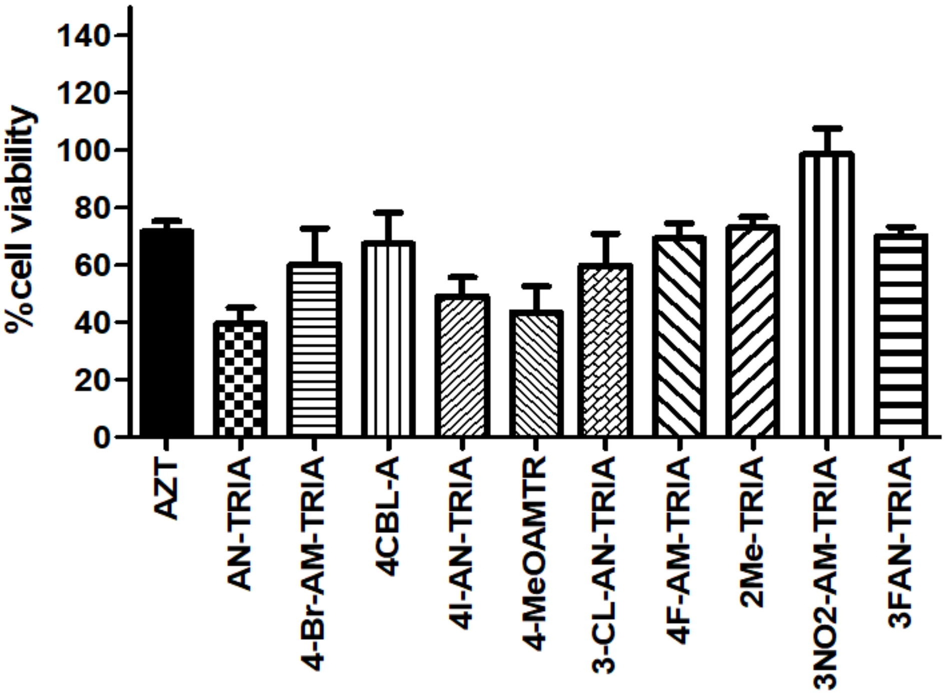

Keys: AN-Tria (**11a**); 3CL-AN-TRIA (**11e**); 4F-AN-TRIA (**11d**); 2MeO-TRIA (**11f**); $3NO_2$-AM-TRIA (**11i**); 3F-AN-TRIA (**11h**); 4BR-AM-TRIA (**11b**); 4CBL-A (**11g**); 4-I-AN-TRIA (**11c**); 4-MeOTRI (**11j**).

4-Azido-7-chloroquinoline (7)

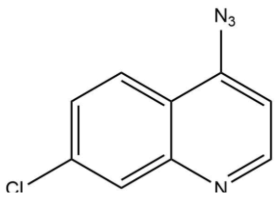

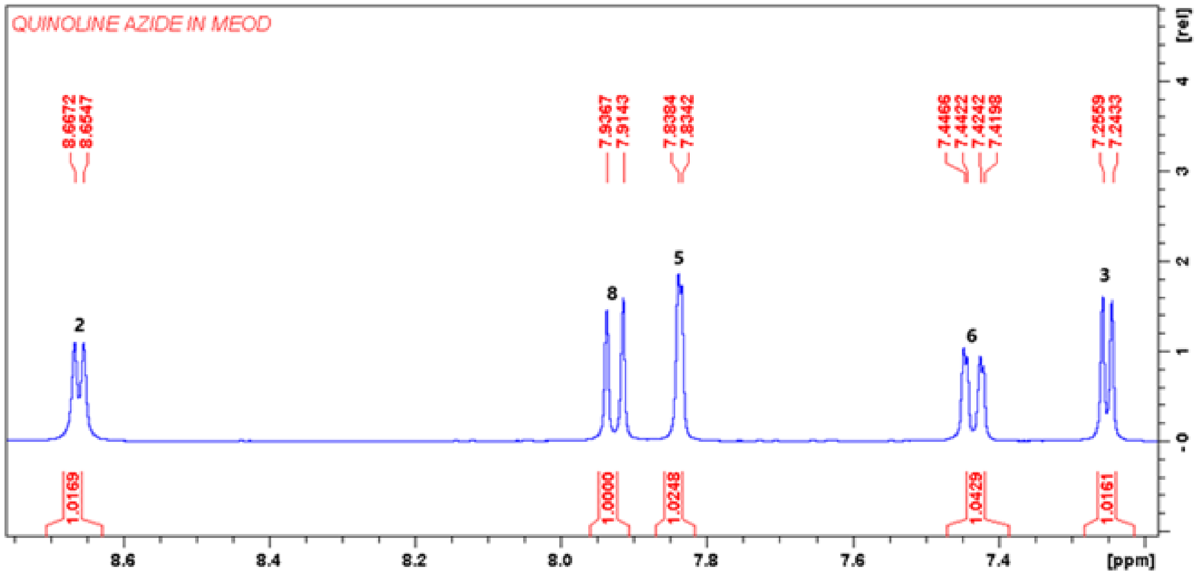

N-2-propyl-1-yl-benzanamine (5a)

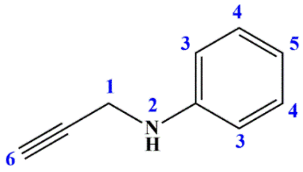

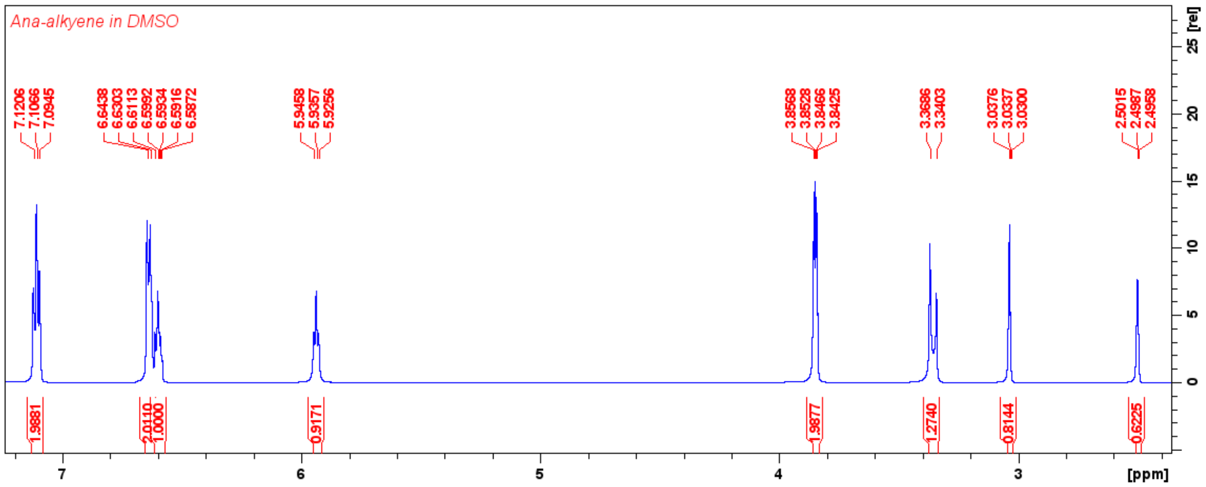

<u>1-(7-Chloro-4-quinolinyl)-1*H*-1,2,3,-triazole-4-methanamine (11a)</u>:

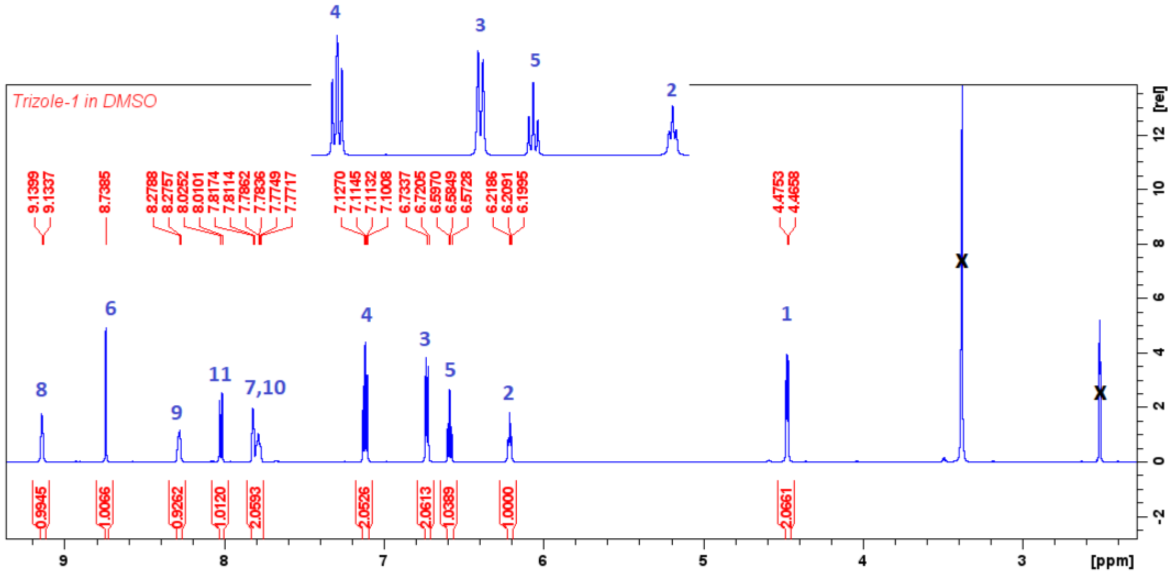

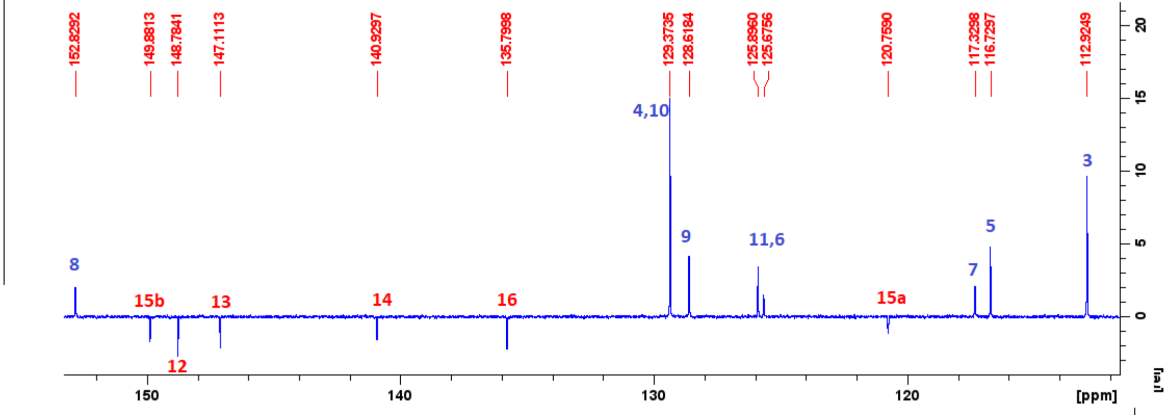

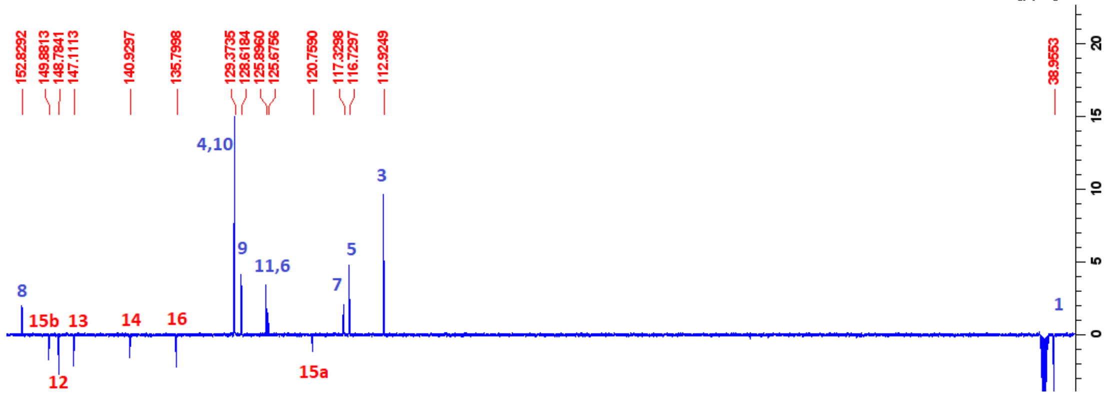

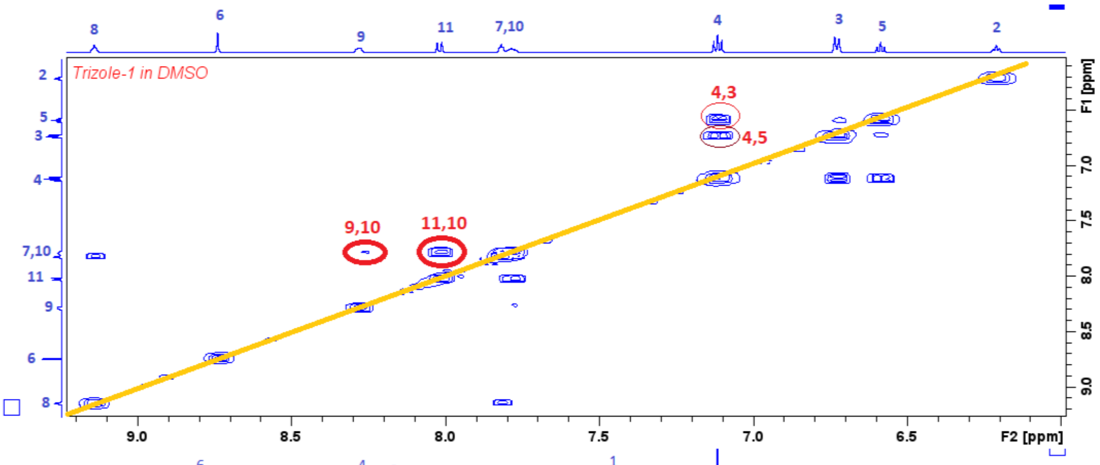
Trizole-1 in DMSO
4,3
4,5
9,10
11,10
F2 [ppm]
F1 [ppm]

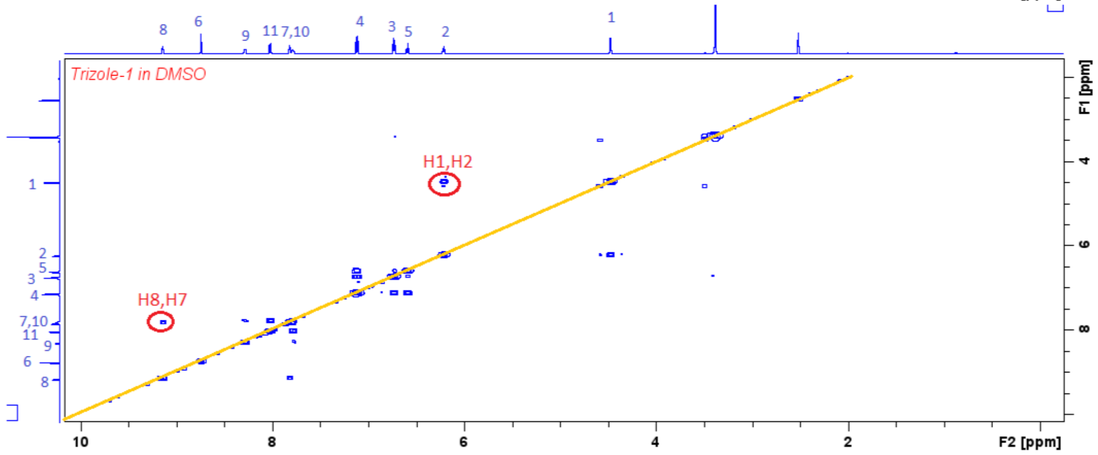
Trizole-1 in DMSO
H1,H2
H8,H7
F2 [ppm]
F1 [ppm]

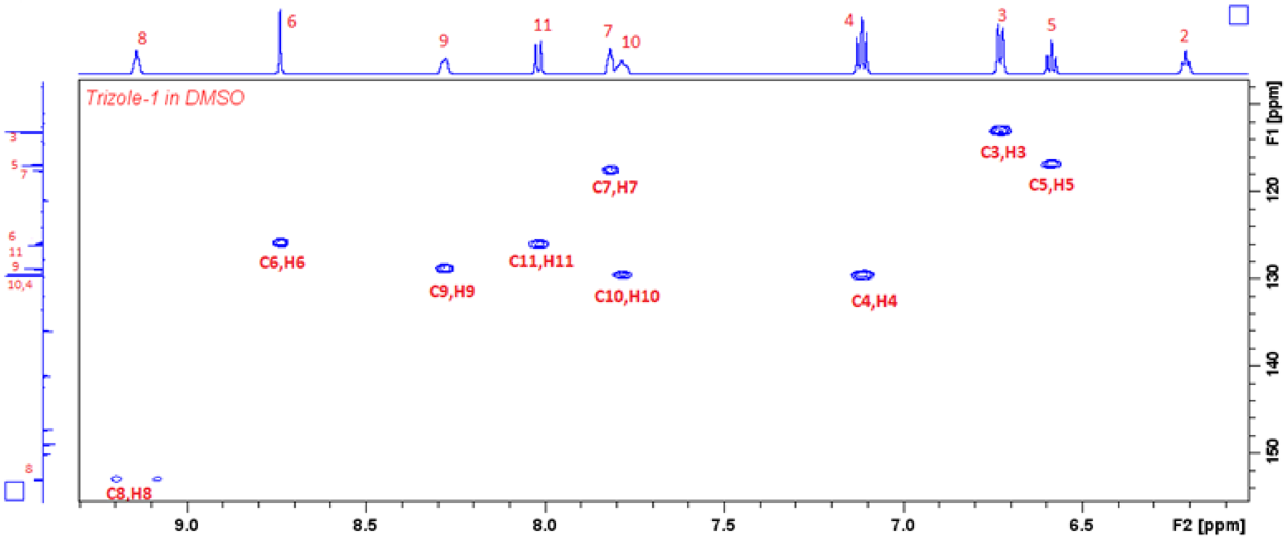
Trizole-1 in DMSO
C3,H3
C5,H5
C7,H7
C6,H6
C11,H11
C9,H9
C10,H10
C4,H4
C8,H8
F2 [ppm]
F1 [ppm]

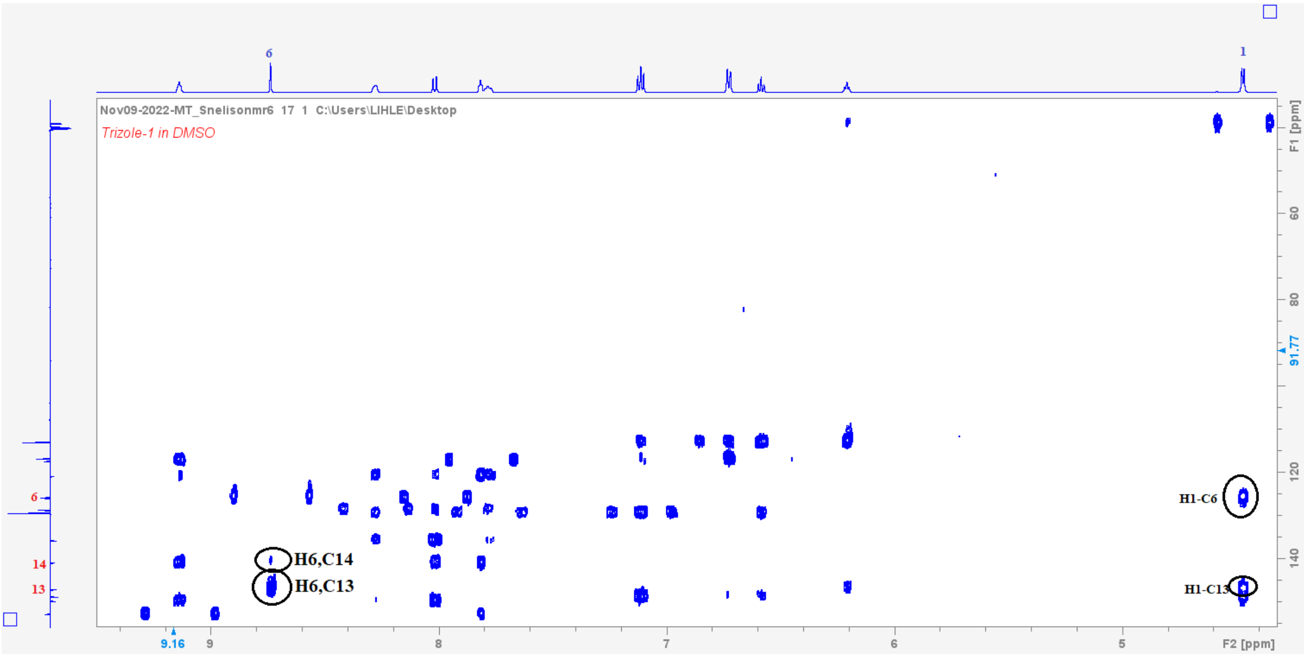
Nov09-2022-MT_Snelisonmr6 17 1 C:\Users\LIHLE\Desktop
Trizole-1 in DMSO
6
1
H6,C14
H6,C13
H1-C6
H1-C13
14
13
9.16
9
8
7
6
5
F2 [ppm]
F1 [ppm]
60
80
91.77
120
140

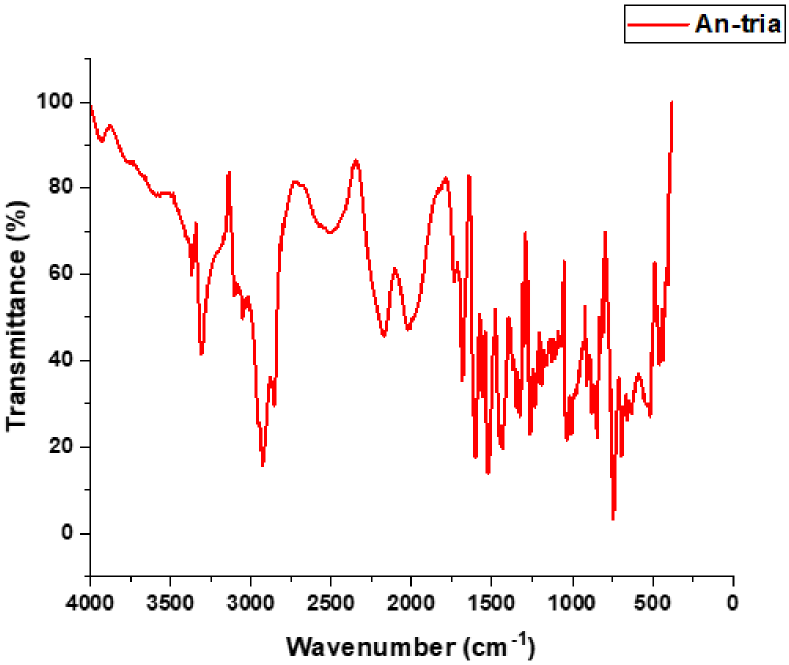
An-tria
Transmittance (%)
100
80
60
40
20
0
4000
3500
3000
2500
2000
1500
1000
500
0
Wavenumber (cm^{-1})

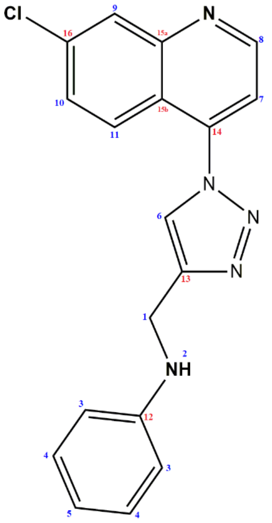
Cl
N
N
N
N
NH

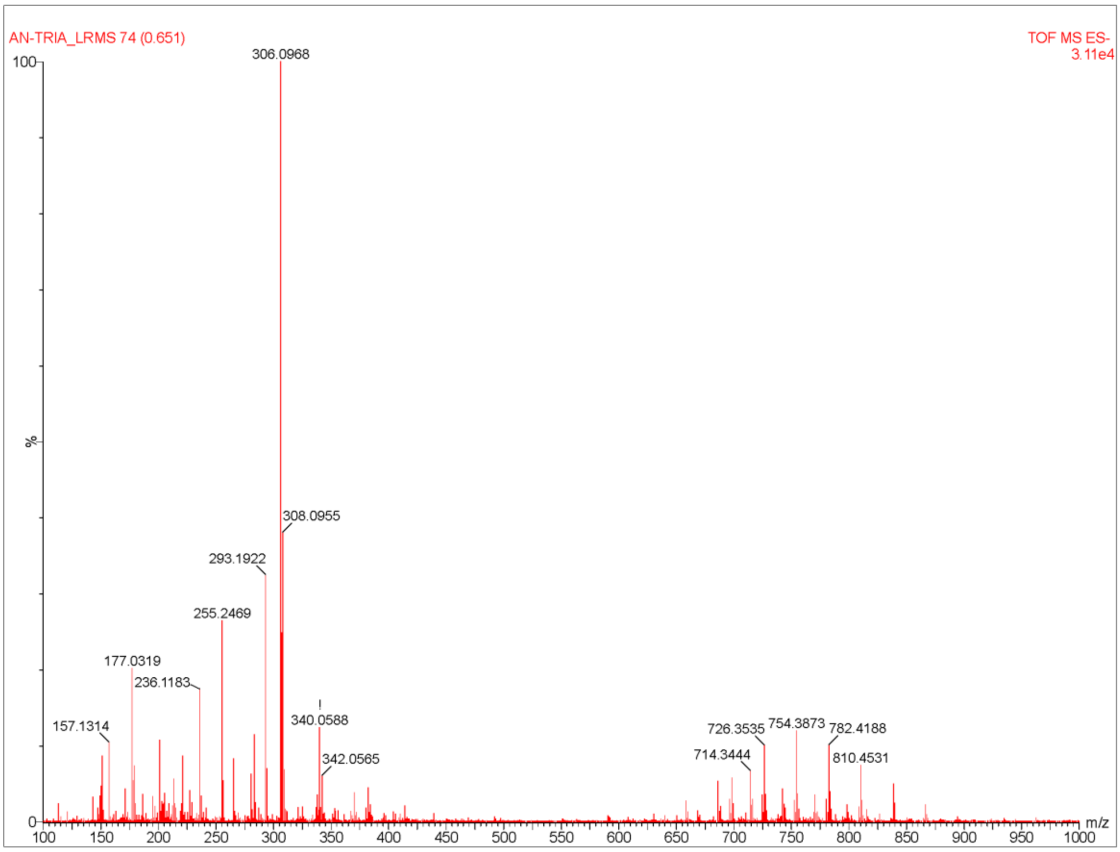

(1-(7-Chloro-4-quinolinyl)-1*H*-1,2,3-triazole-4-methyl)-4-bromoaniline (11b):

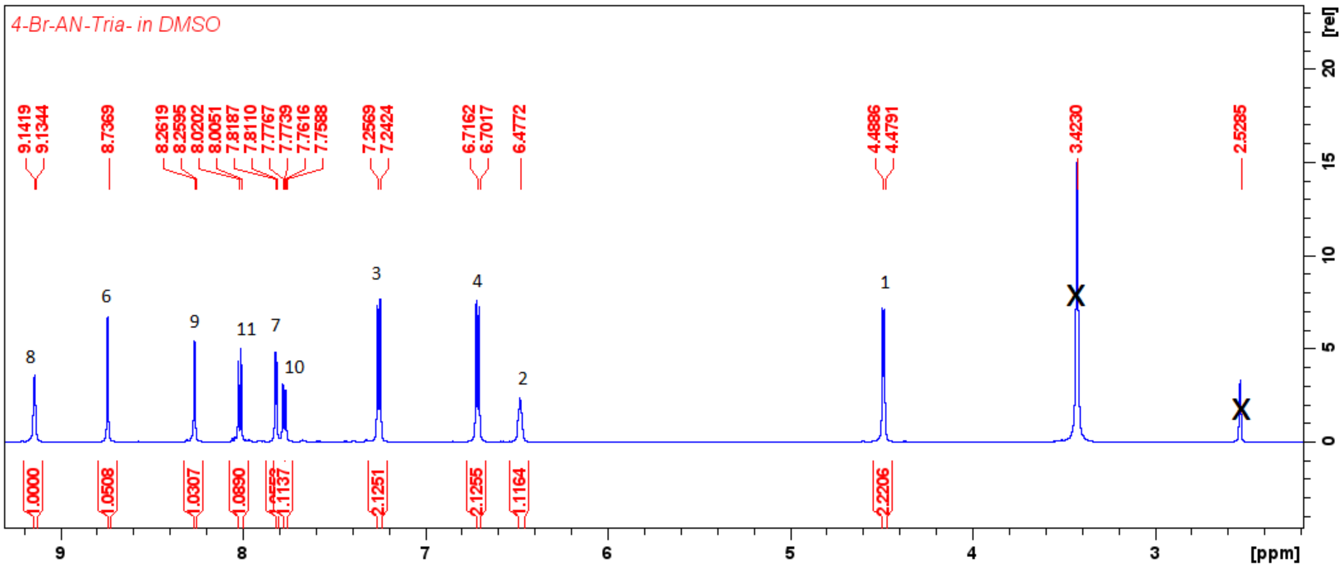

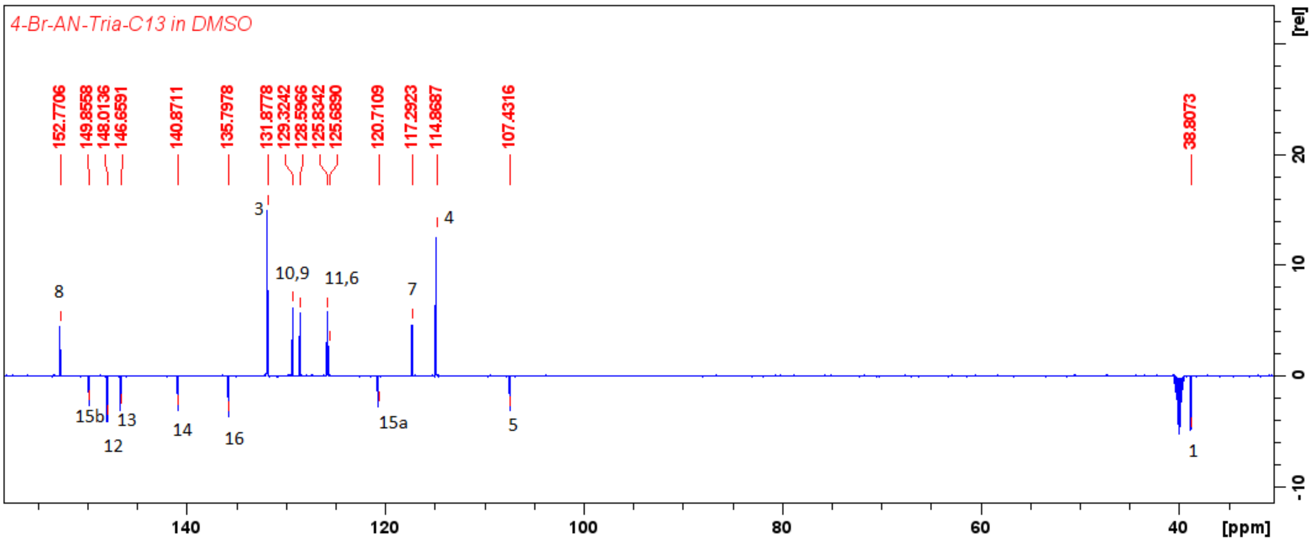
4-Br-AN-Tria-C13 in DMSO
152.7706
149.8558
148.0136
146.6591
140.8711
135.7978
131.8778
129.3242
128.5966
125.8342
125.6890
120.7109
117.2923
114.8687
107.4316
38.8073
8
3
10,9
11,6
7
4
15b
13
12
14
16
15a
5
1
[rel]
[ppm]

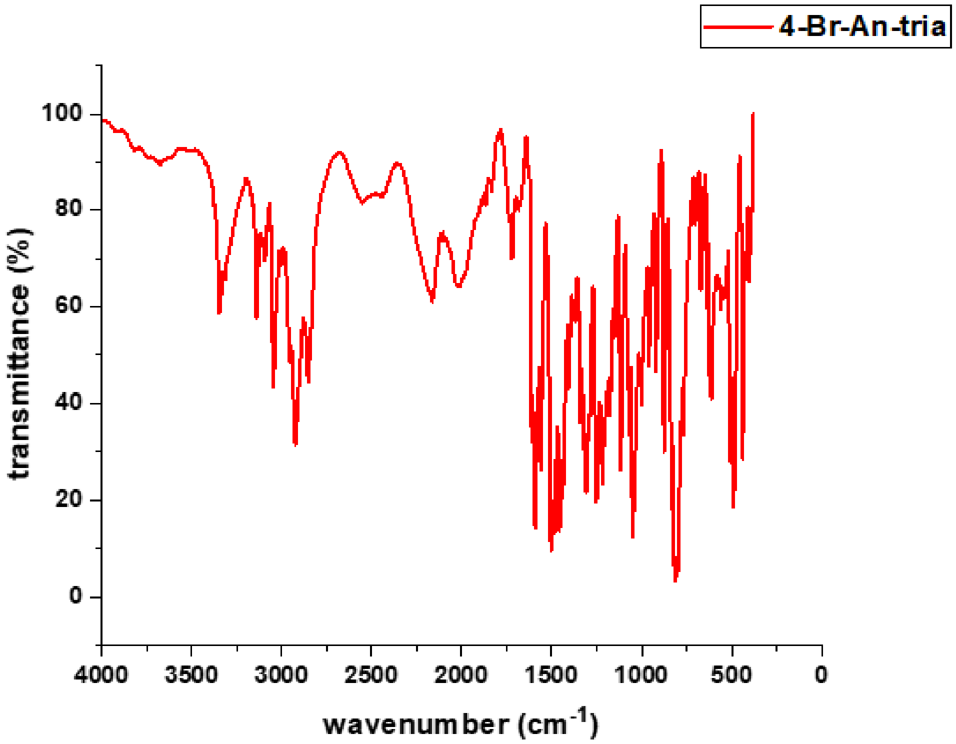
4-Br-An-tria
transmittance (%)
wavenumber (cm-1)

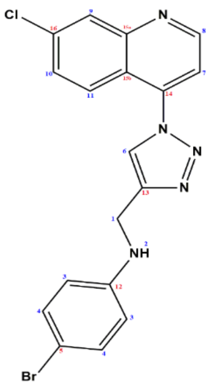
Cl
N
N
N
N
NH
Br

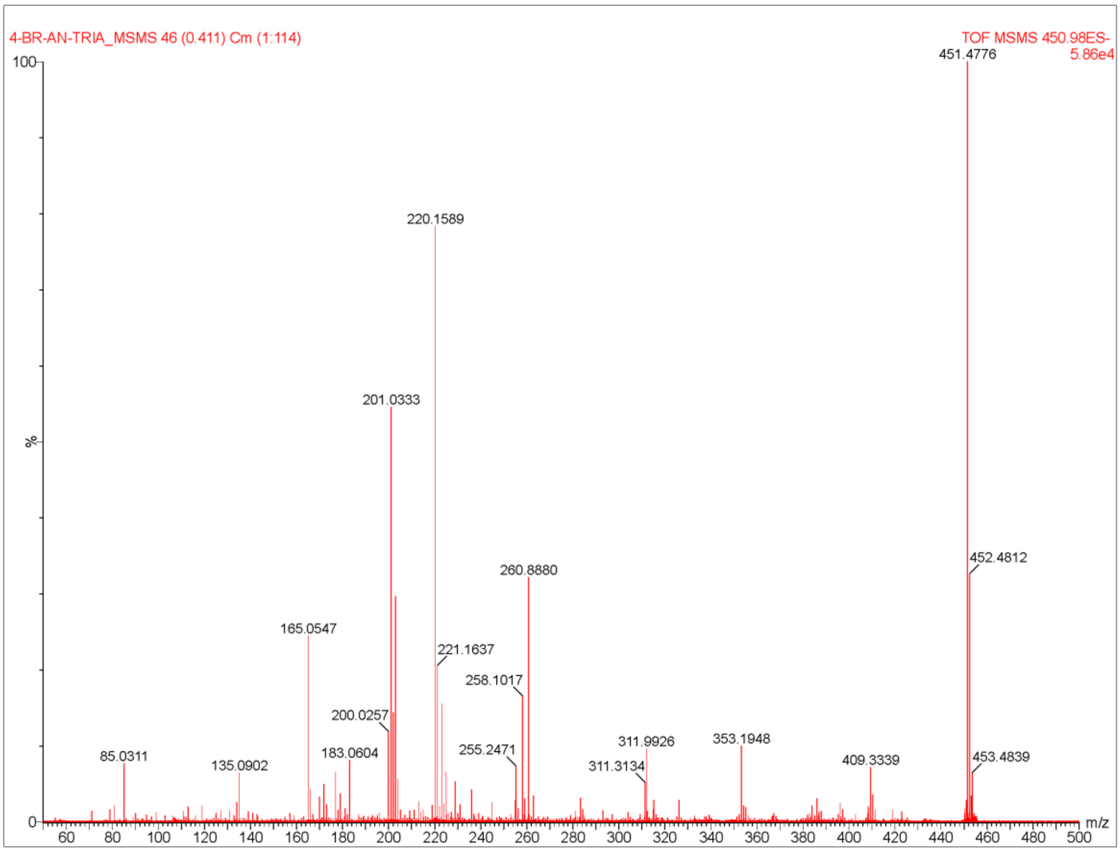

(1-(7-Chloro-4-quinolinyl)-1*H*-1,2,3-triazole-4-methyl)-4-iodoaniline (11c):

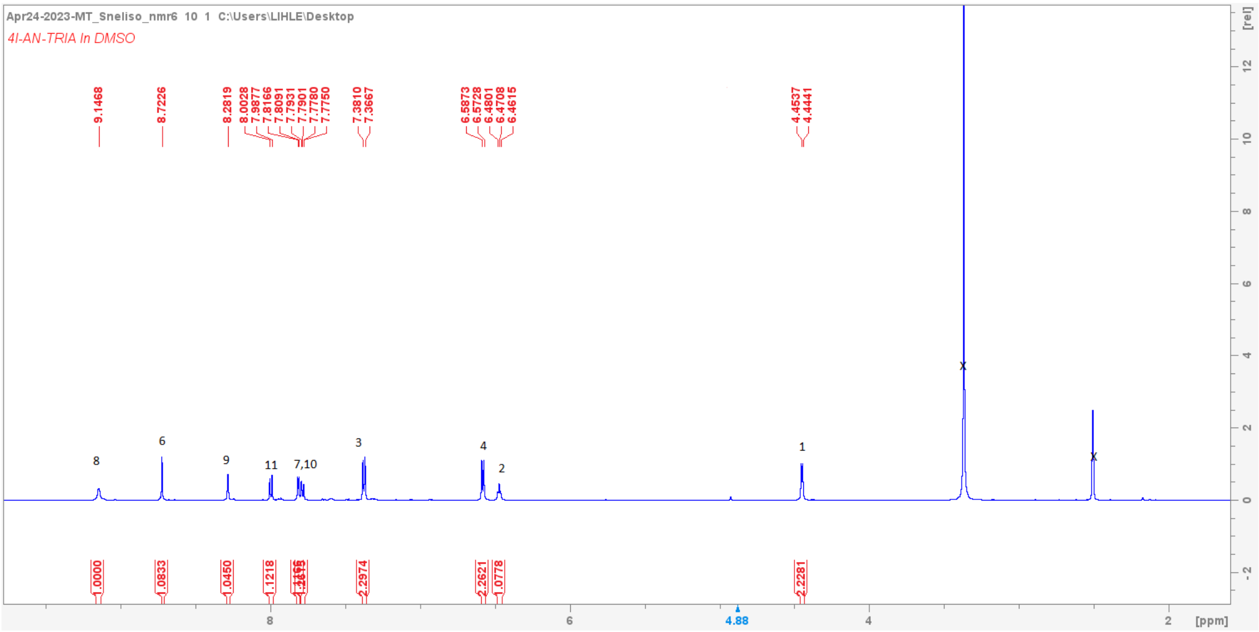

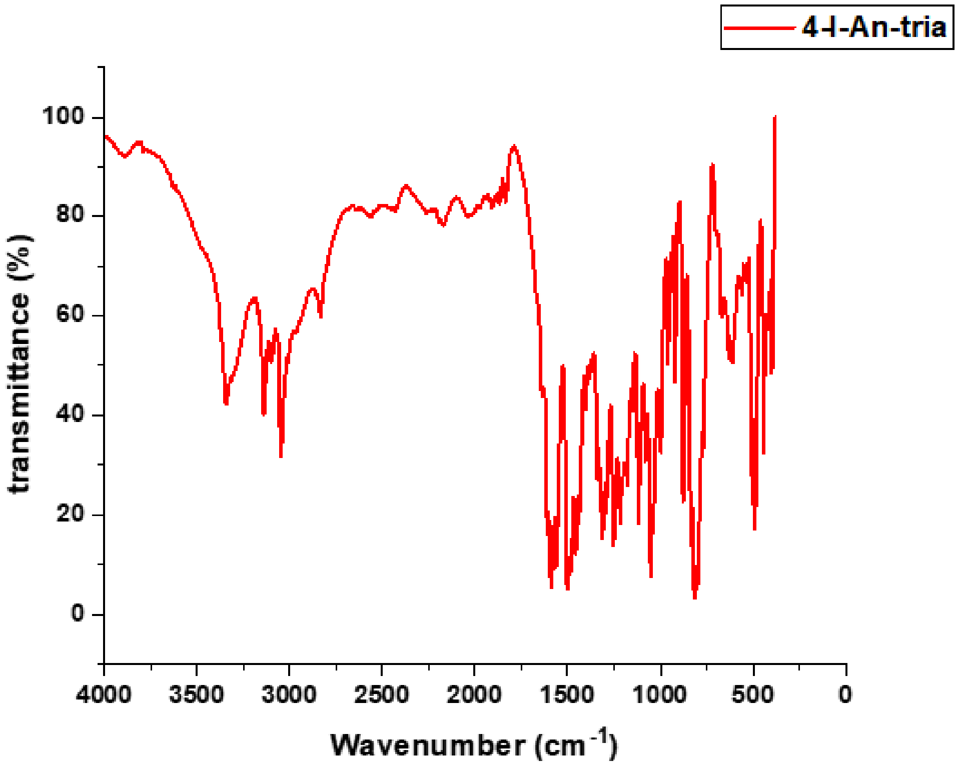
4-I-An-tria
transmittance (%)
100
80
60
40
20
0
4000
3500
3000
2500
2000
1500
1000
500
0
Wavenumber (cm^{-1})

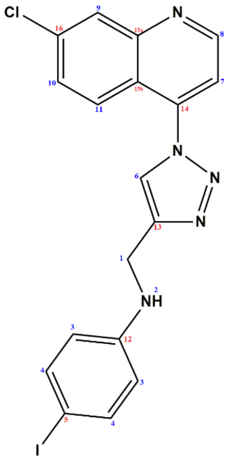
Cl
N
N
N
N
NH
I

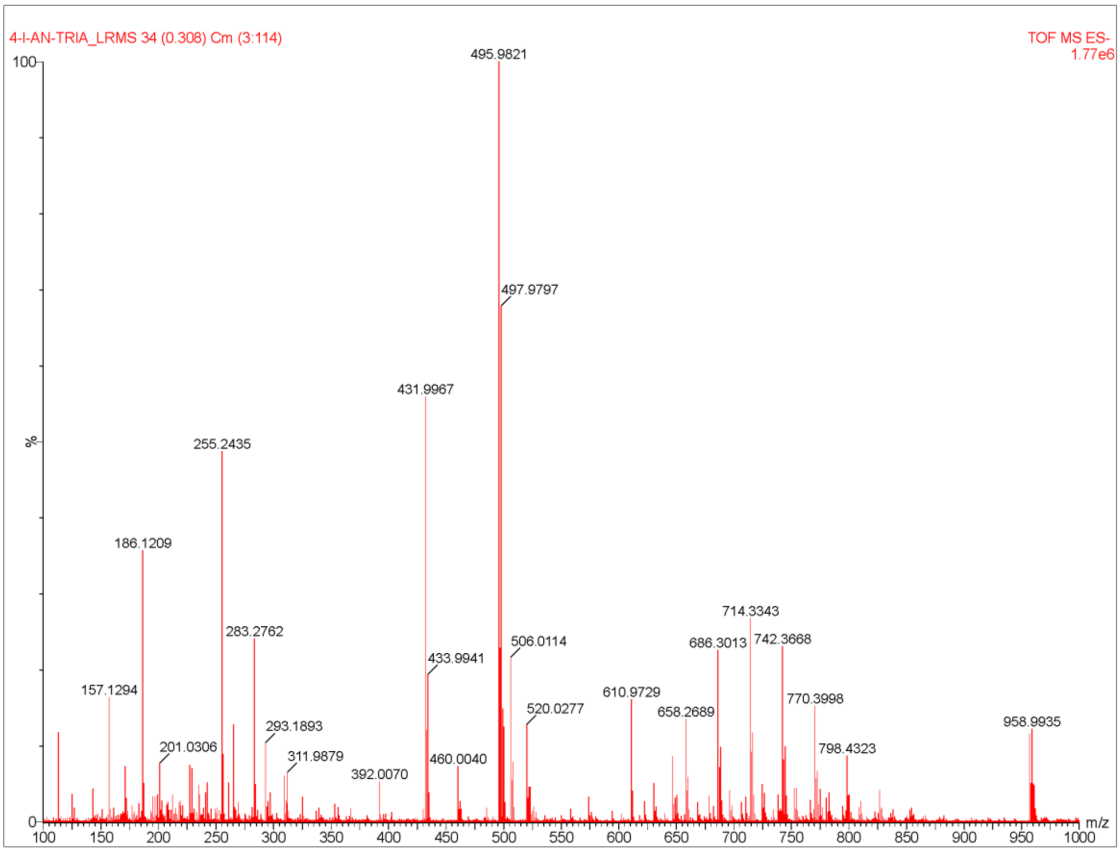
4-I-AN-TRIA_LRMS 34 (0.308) Cm (3:114)
TOF MS ES-
1.77e6
100
%
0
495.9821
497.9797
431.9967
255.2435
186.1209
283.2762
433.9941
506.0114
714.3343
686.3013
742.3668
157.1294
610.9729
658.2689
770.3998
520.0277
293.1893
201.0306
311.9879
460.0040
392.0070
798.4323
958.9935
m/z
100
150
200
250
300
350
400
450
500
550
600
650
700
750
800
850
900
950
1000

(1-(7-Chloro-4-quinolinyl)-1*H*-1,2,3-triazole-4-methyl)-4-floroaniline (11d):

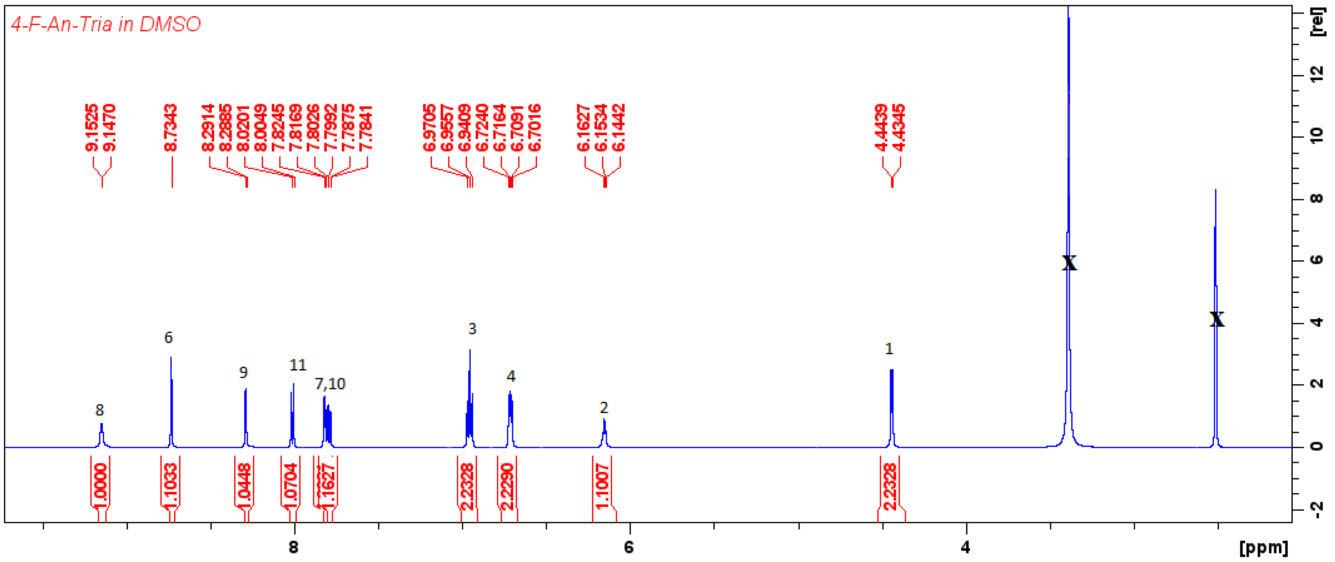

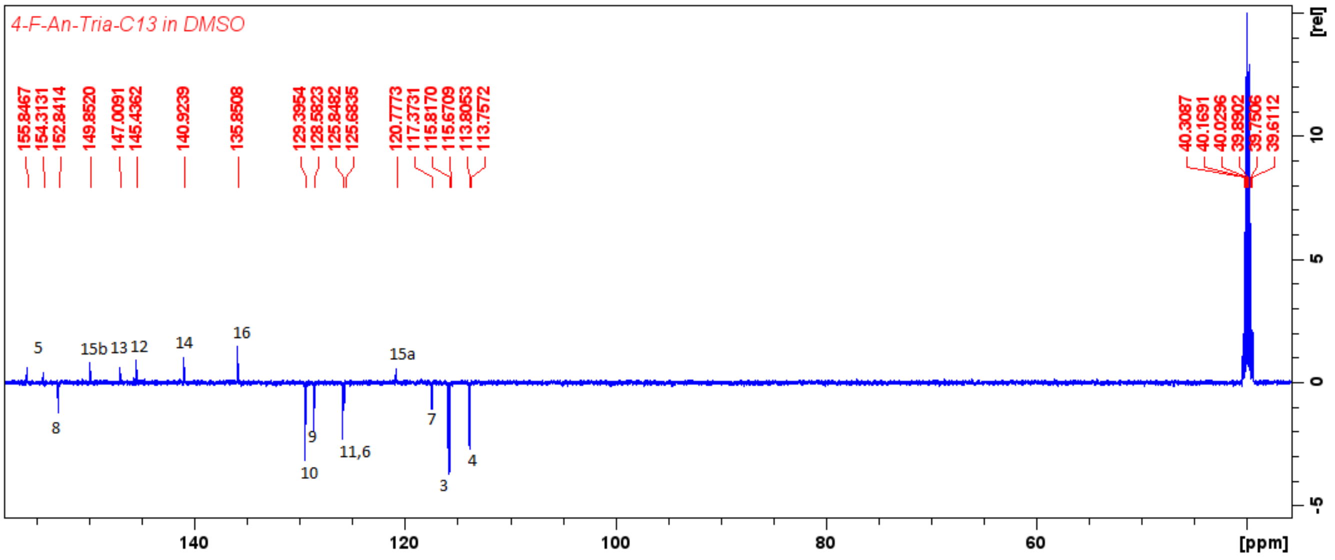

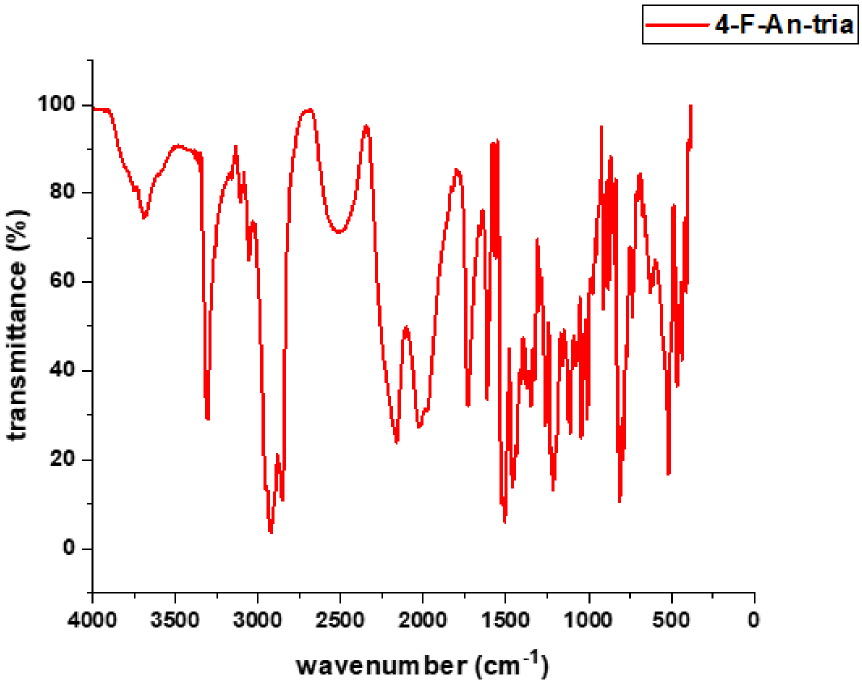
4-F-An-tria
transmittance (%)
wavenumber (cm-1)
100
80
60
40
20
0
4000
3500
3000
2500
2000
1500
1000
500
0

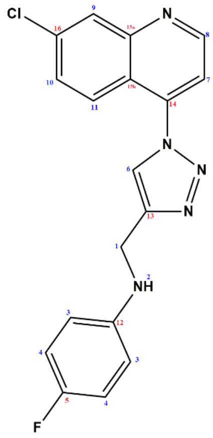
Cl
N
N
N
N
NH
F

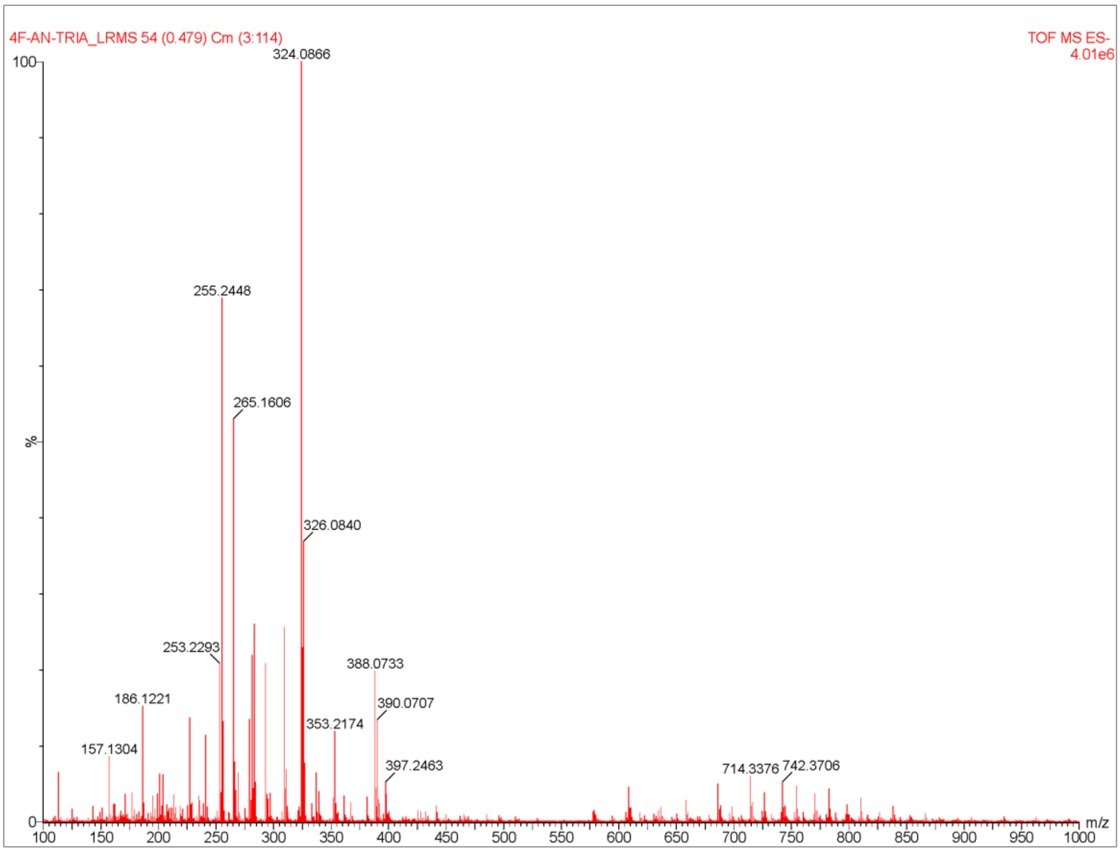
4F-AN-TRIA_LRMS 54 (0.479) Cm (3:114)
TOF MS ES-
4.01e6
100
%
0
324.0866
255.2448
265.1606
326.0840
253.2293
388.0733
186.1221
390.0707
353.2174
157.1304
397.2463
714.3376
742.3706
m/z
100
150
200
250
300
350
400
450
500
550
600
650
700
750
800
850
900
950
1000

Compound 11d F-H and F-C couplings expanded regions:

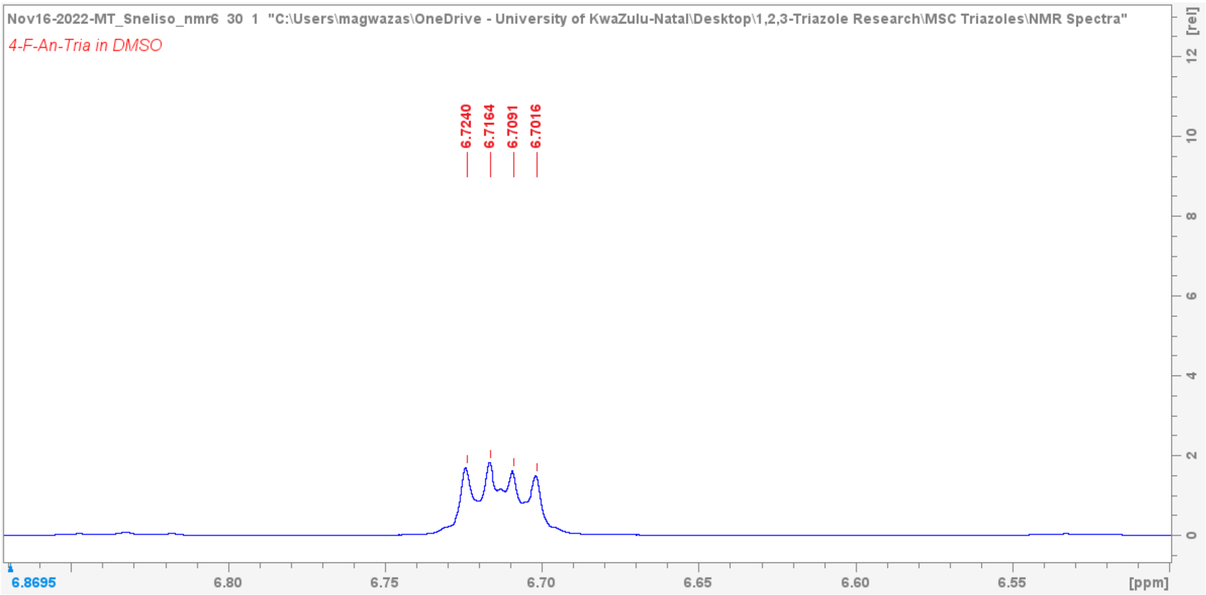

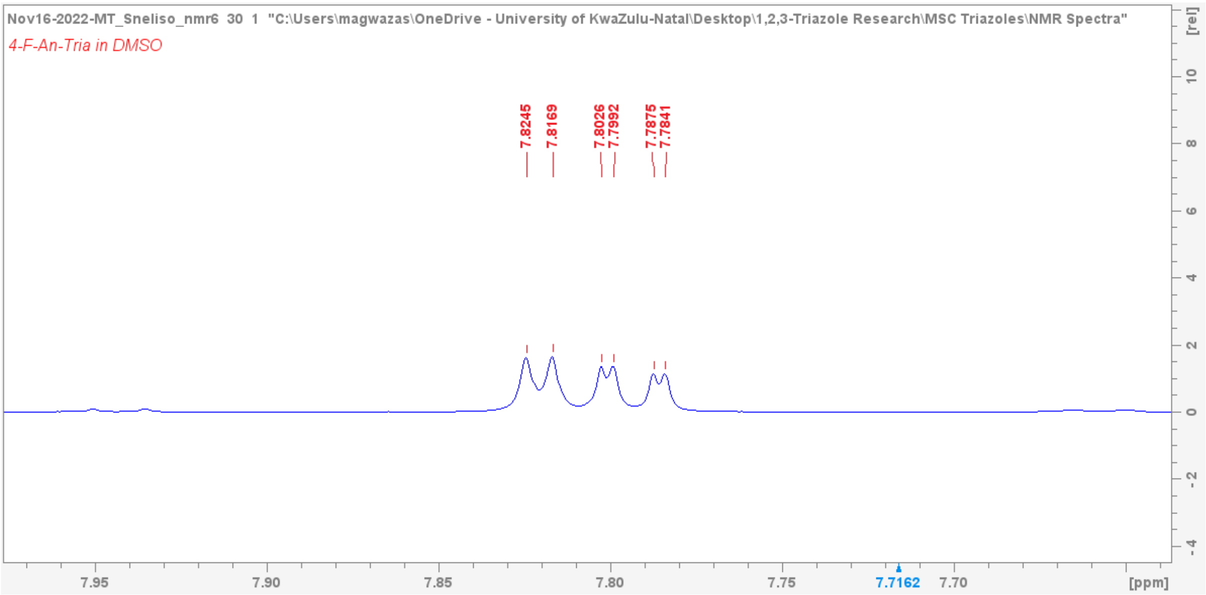

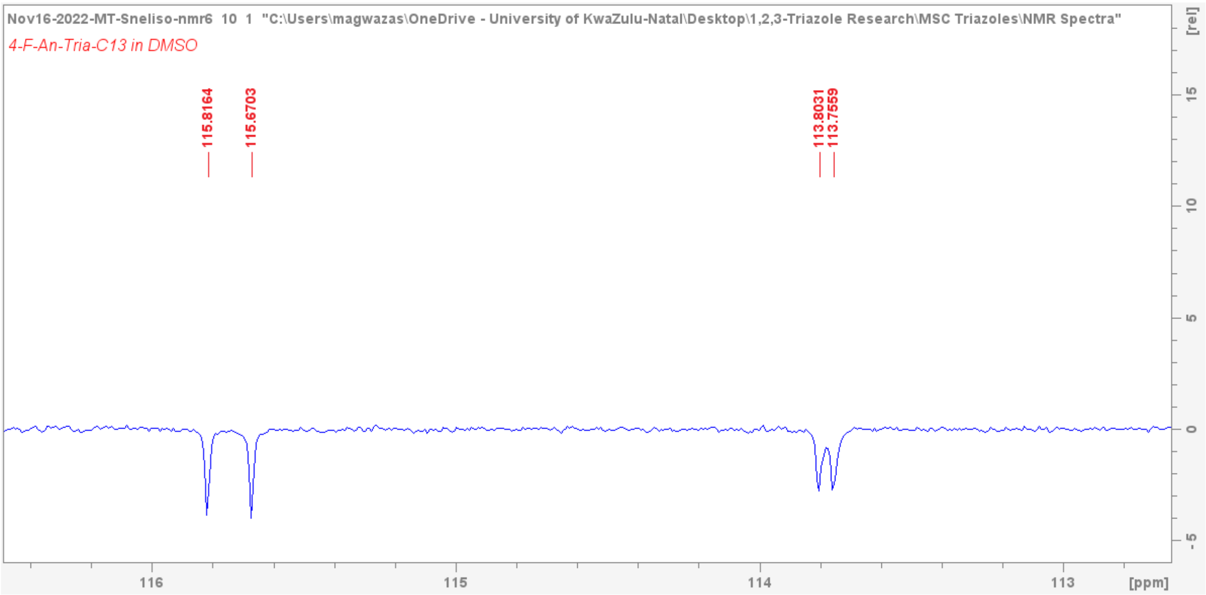
Nov16-2022-MT-Sneliso-nmr6 10 1 "C:\Users\magwazas\OneDrive - University of KwaZulu-Natal\Desktop\1,2,3-Triazole Research\MSC Triazoles\NMR Spectra"
4-F-An-Tria-C13 in DMSO
115.8164
115.6703
113.8031
113.7559
[rel]
15
10
5
0
-5
116
115
114
113
[ppm]

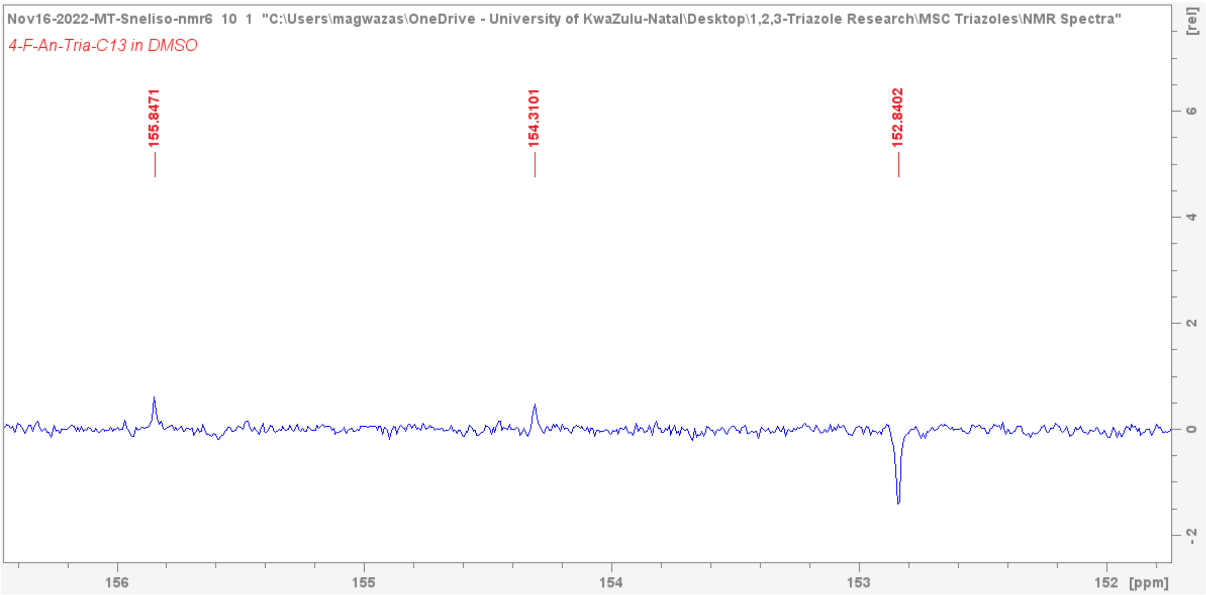
Nov16-2022-MT-Sneliso-nmr6 10 1 "C:\Users\magwazas\OneDrive - University of KwaZulu-Natal\Desktop\1,2,3-Triazole Research\MSC Triazoles\NMR Spectra"
4-F-An-Tria-C13 in DMSO
155.8471
154.3101
152.8402
[rel]
6
4
2
0
-2
156
155
154
153
152
[ppm]

(1-(7-Chloro-4-qiunolinyl)-1*H*-1,2,3-triazole-4-nethyl)-3-chloroaniline (11e):

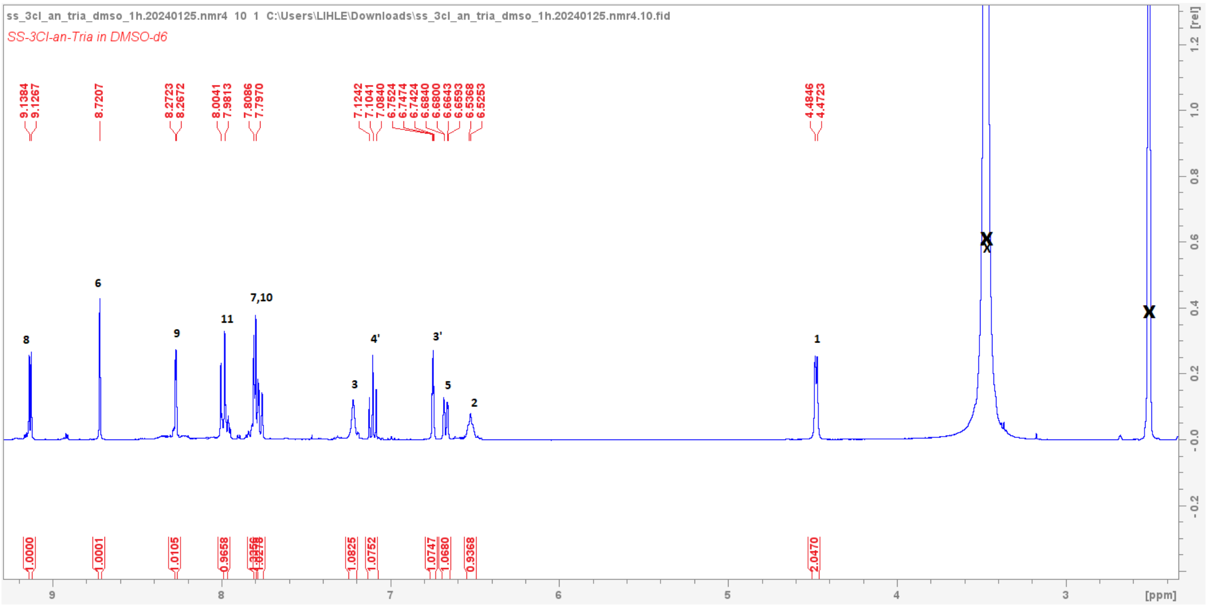

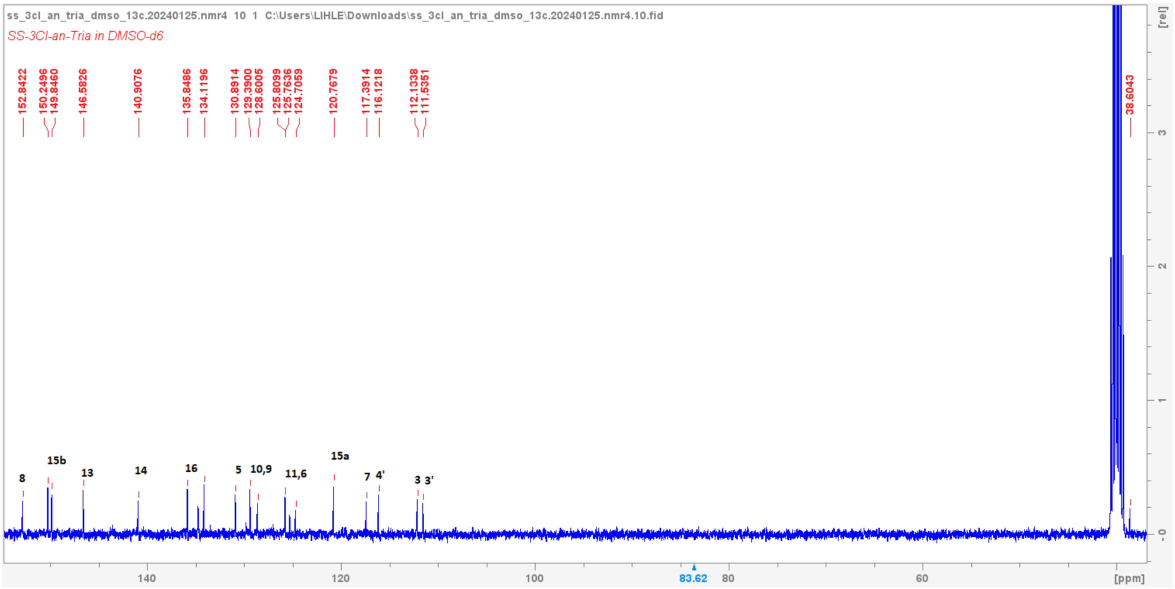

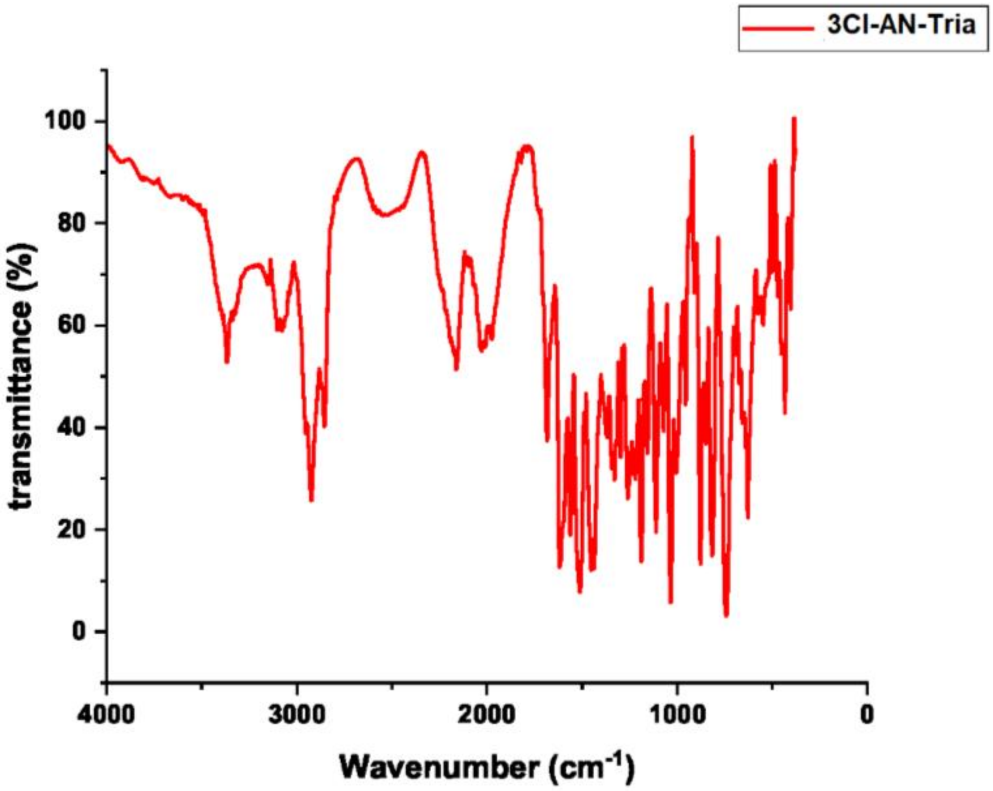
3Cl-AN-Tria
transmittance (%)
100
80
60
40
20
0
4000
3000
2000
1000
0
Wavenumber (cm-1)

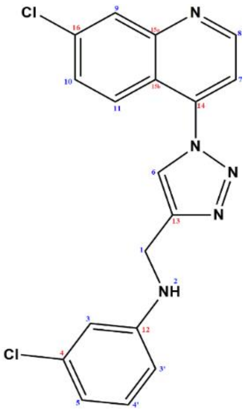
Cl
N
N
N
N
NH
Cl

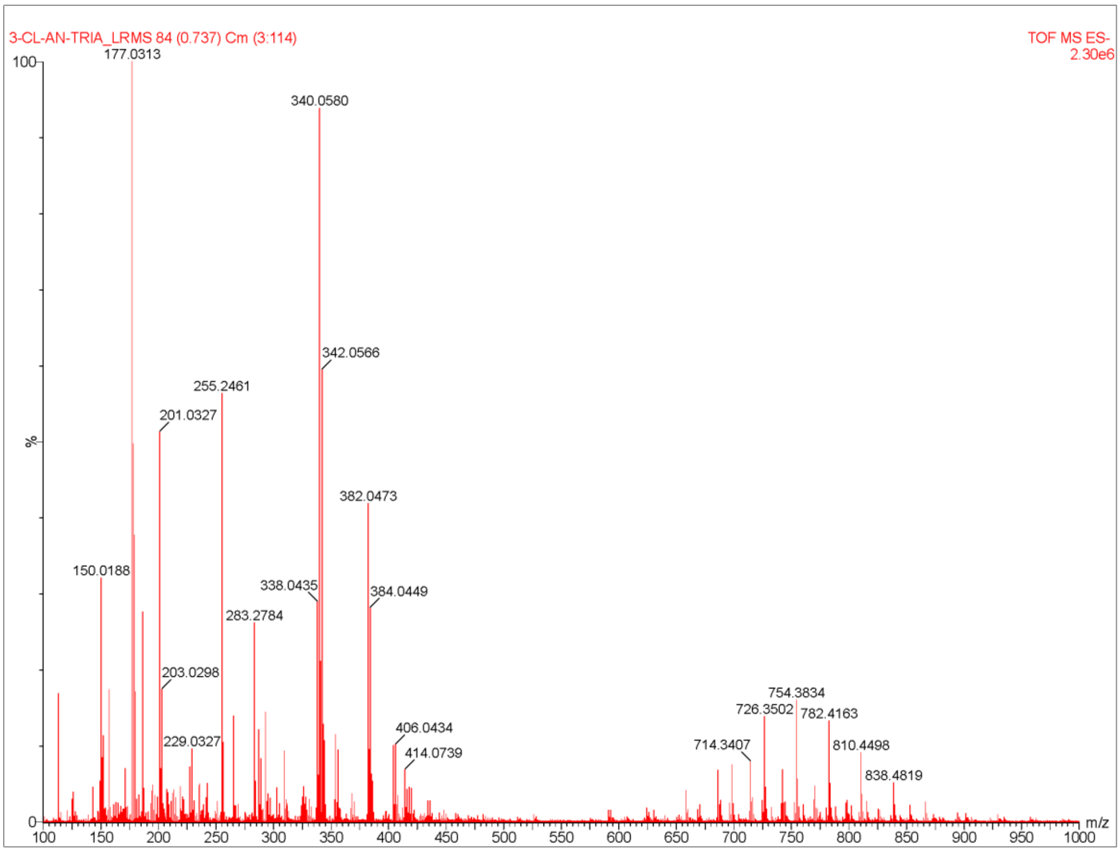
3-CL-AN-TRIA_LRMS 84 (0.737) Cm (3:114)
TOF MS ES-
2.30e6
100
%
0
177.0313
340.0580
342.0566
255.2461
201.0327
382.0473
150.0188
338.0435
384.0449
283.2784
203.0298
229.0327
406.0434
414.0739
754.3834
726.3502
782.4163
714.3407
810.4498
838.4819
m/z
100
150
200
250
300
350
400
450
500
550
600
650
700
750
800
850
900
950
1000

(1-(7-Chloro-4-quinolinyl)-1*H*-1,2,3-triazole-4-methyl)-2-methoxyaniline (11f):

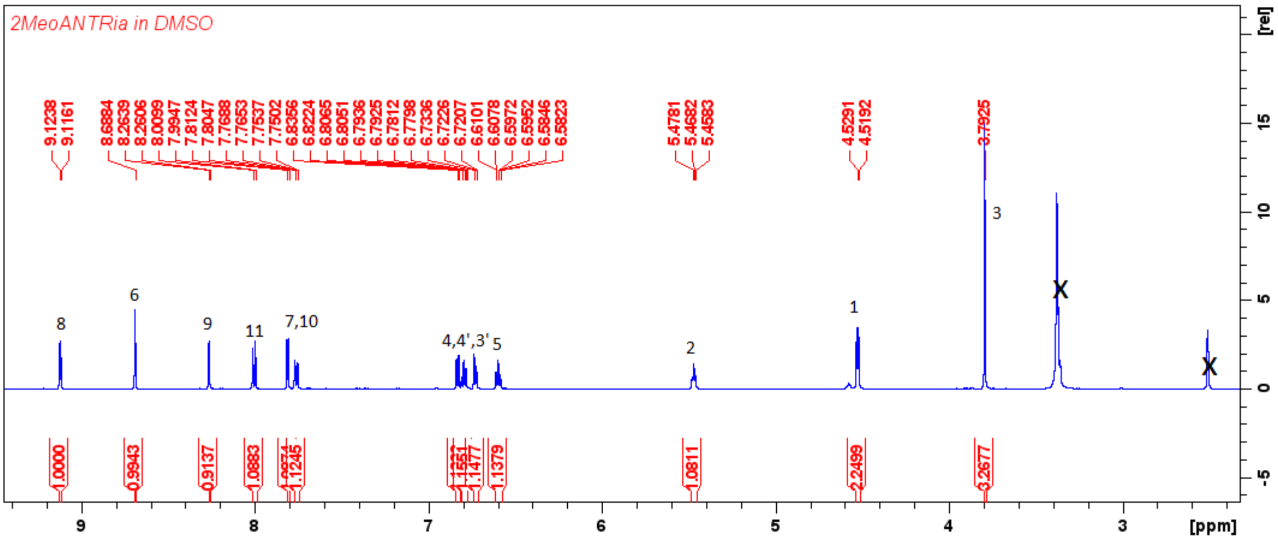

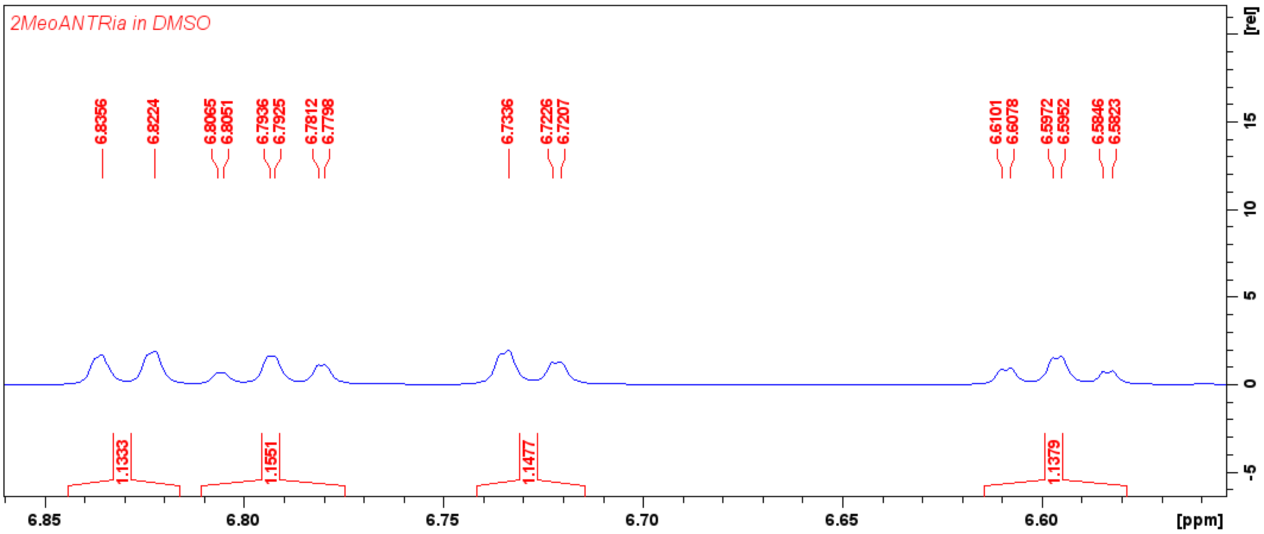

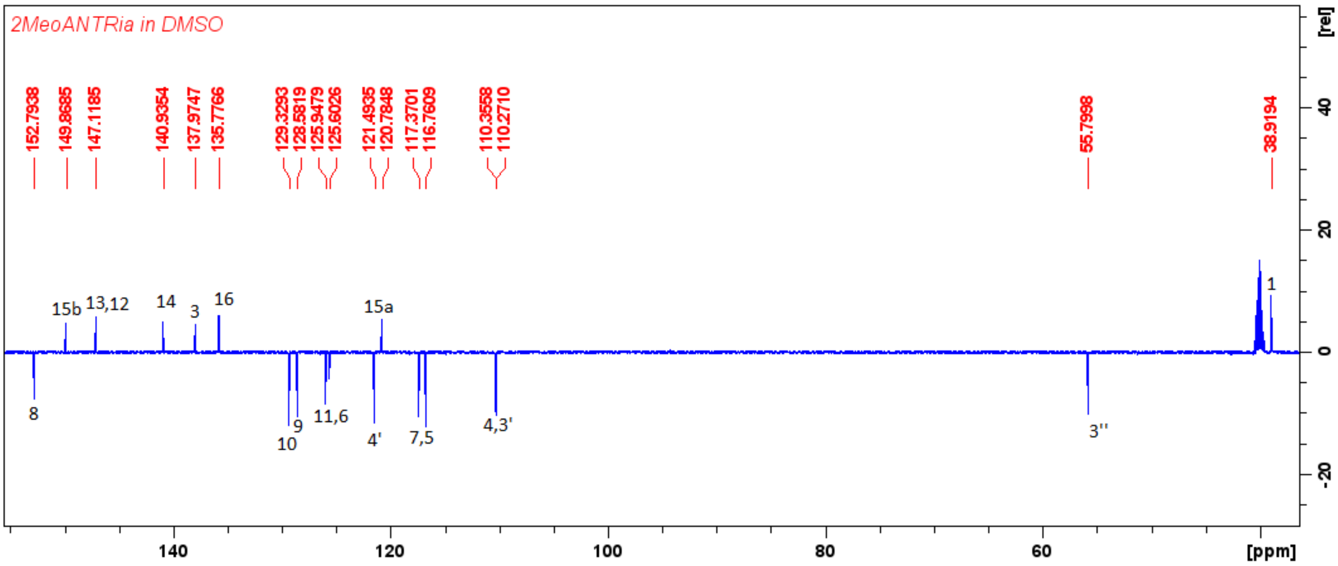

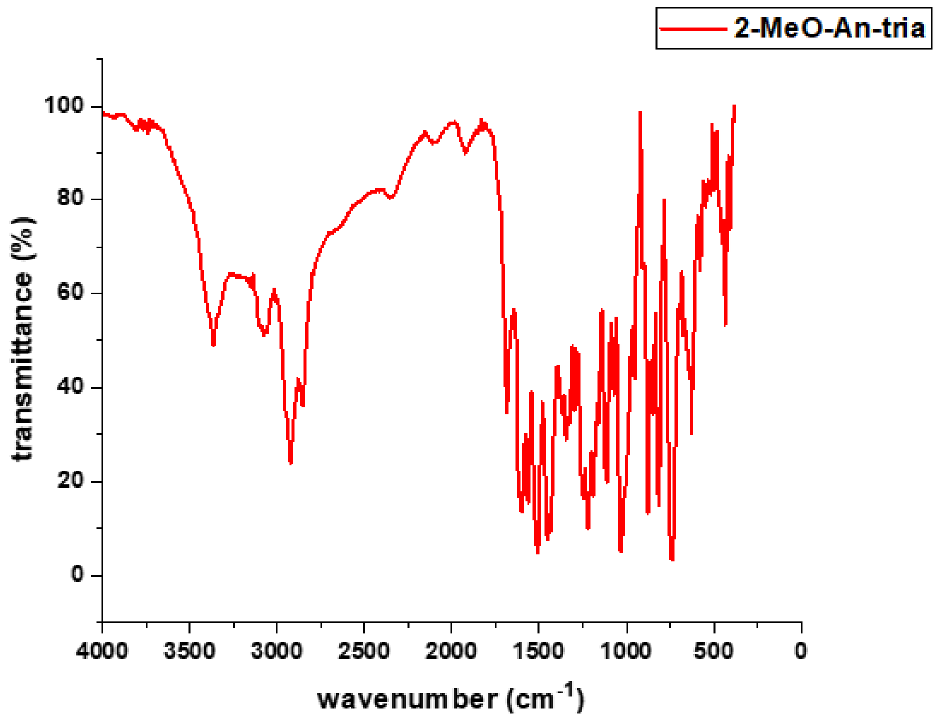
2-MeO-An-tria
transmittance (%)
100
80
60
40
20
0
4000
3500
3000
2500
2000
1500
1000
500
0
wavenumber (cm^{-1})

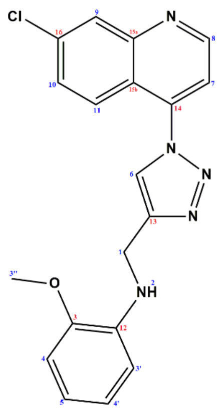
Cl
N
N
N
N
NH
O

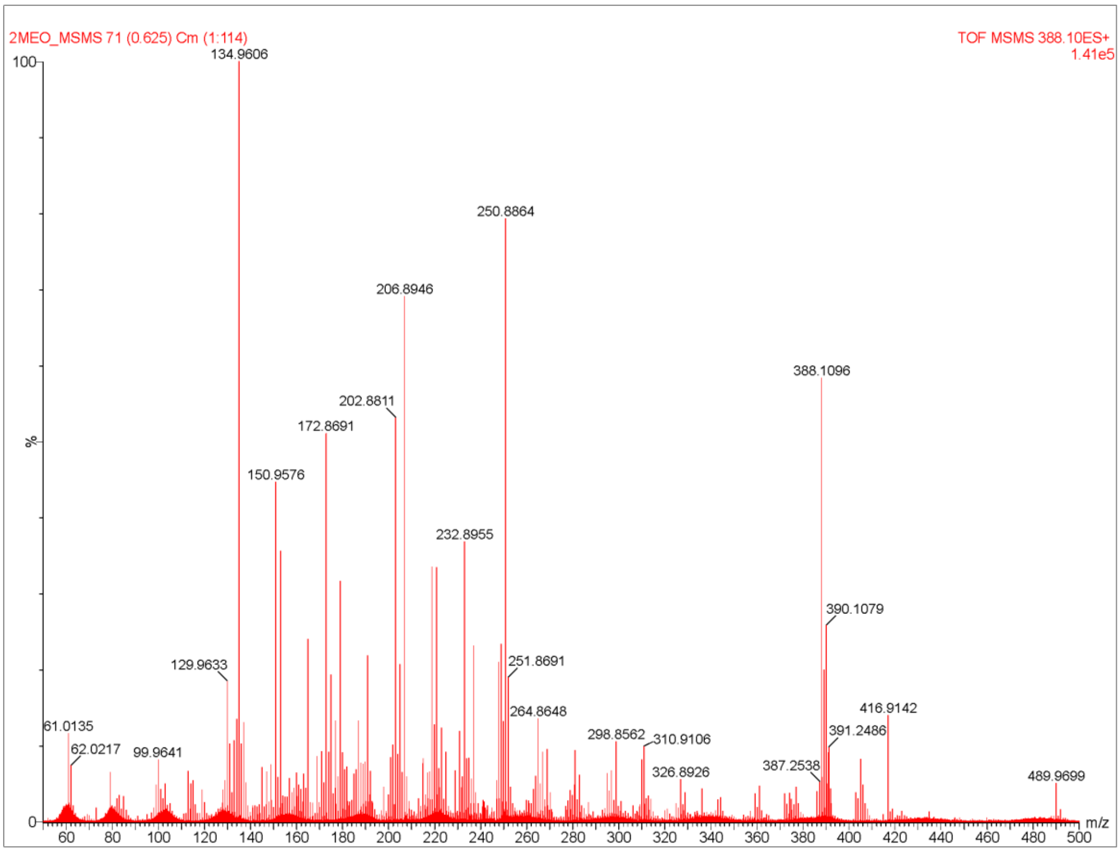
2MEO_MSMS 71 (0.625) Cm (1:114)
TOF MSMS 388.10ES+
1.41e5
100
%
0
134.9606
250.8864
206.8946
388.1096
202.8811
172.8691
150.9576
232.8955
390.1079
129.9633
251.8691
264.8648
416.9142
61.0135
62.0217
99.9641
298.8562
310.9106
391.2486
326.8926
387.2538
489.9699
60
80
100
120
140
160
180
200
220
240
260
280
300
320
340
360
380
400
420
440
460
480
500
m/z

(1-(7-Chloro-4-quinolinyl)-1*H*-1,2,3-triazole-4-methyl)-2-triflouromethylaniline (11g):

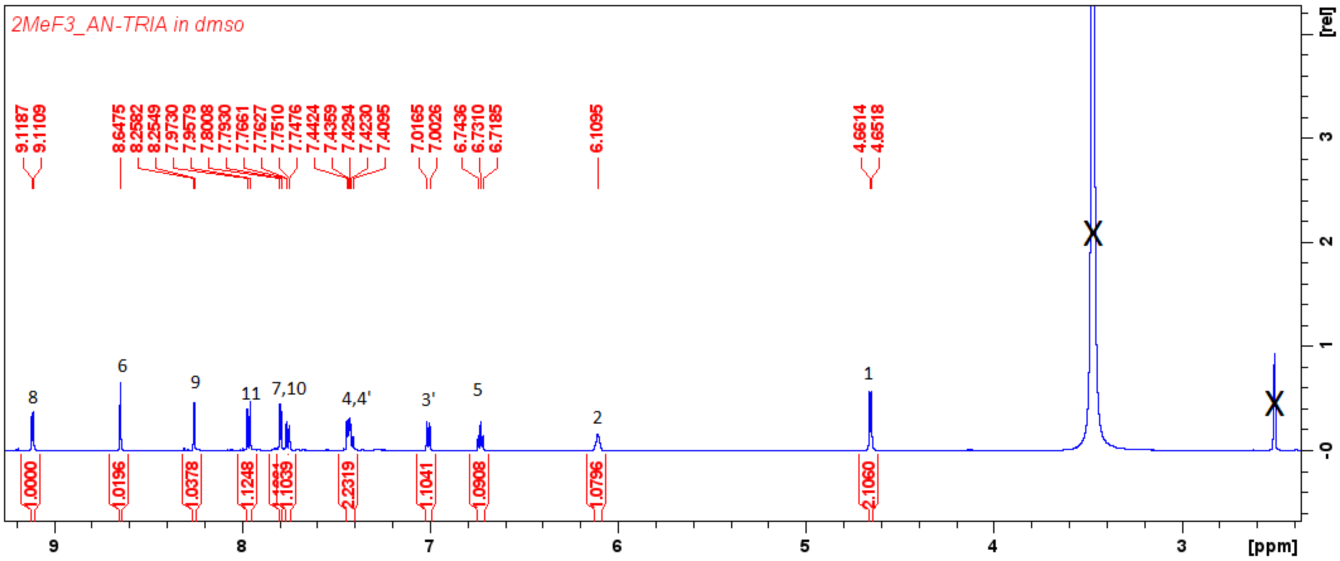

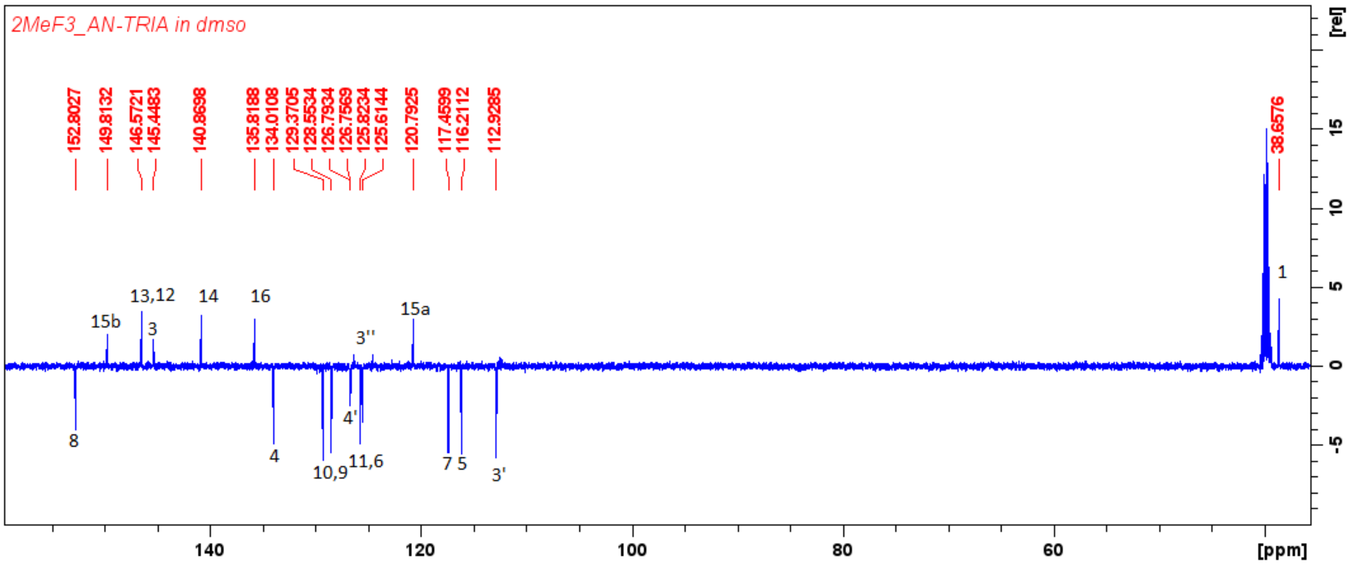

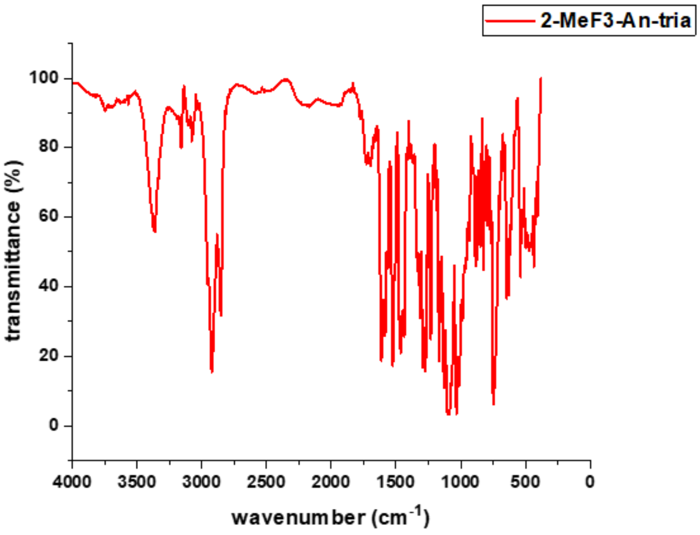

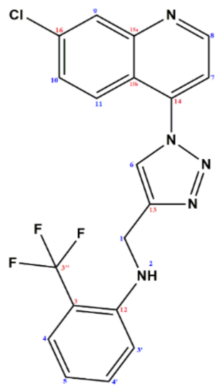

Sample Information

Date Acquired	: 2012/05/24 11:18:25 PM
Sample Type	: Standard
Sample Name	: 11g
Sample ID	: 11g
Data File	: 11g.lcd
Method File	: no column run 2025.lcm
Report Format File	: mass spectrum %.lsr
Tuning File	: 260125.lct

MS Spectrum

R.Time:1.083(Scan#:66)
MassPeaks:2059
Segment 1 - Event 1

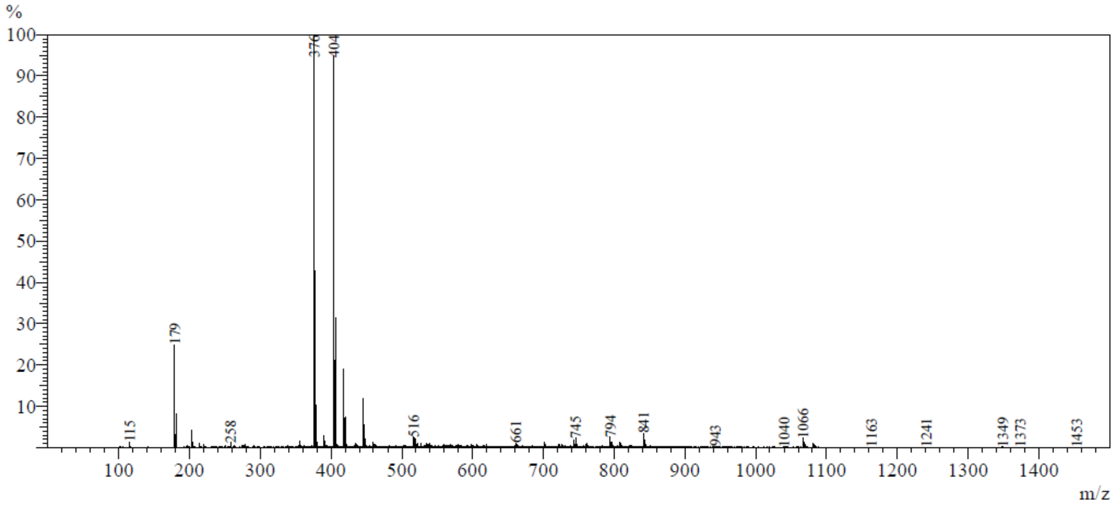

Instrument details: Shimadzu :LCMS-2020, Prominence -i(HPLC)

mobile phase : 100%ACN with 0.1 % formic acid

Flow rate : 0.2ml/min

It was run through a loop(no column.

(1-(7-Chloro-4-quinolinyl)-1*H*-1,2,3-triazole-4-methyl)-3-flouroaniline (11h) :

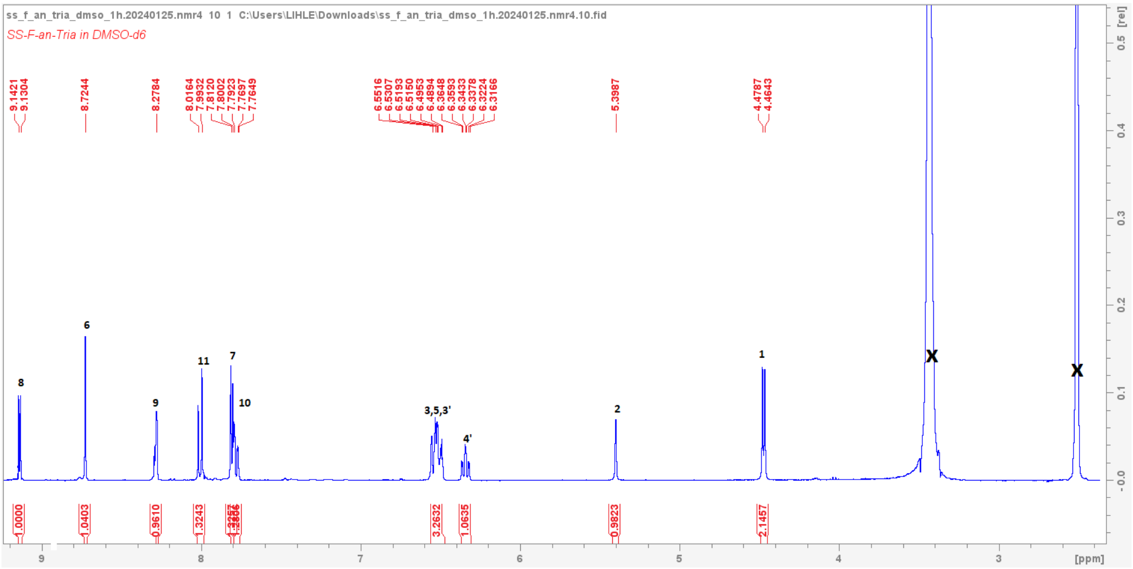

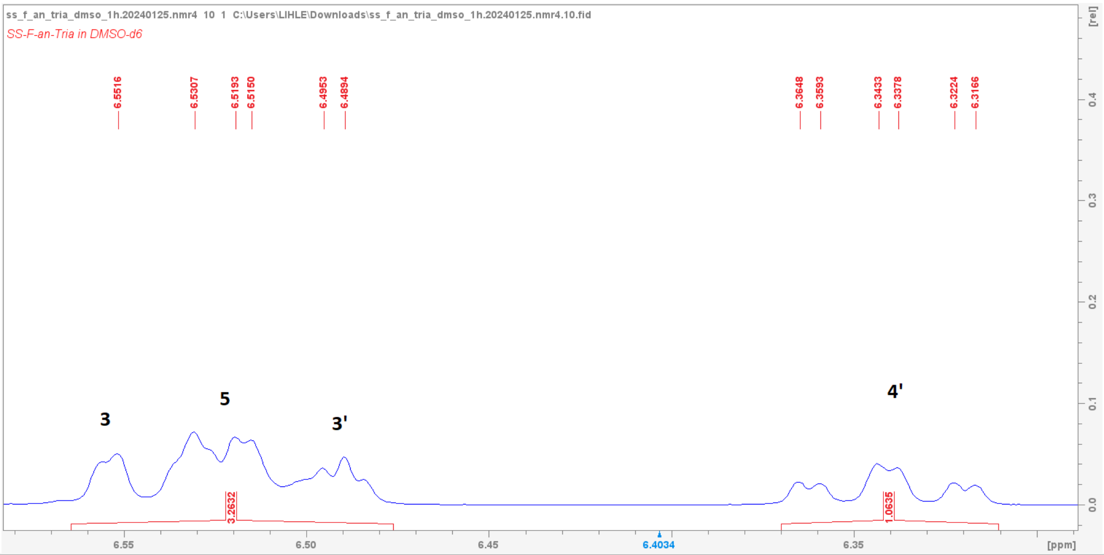

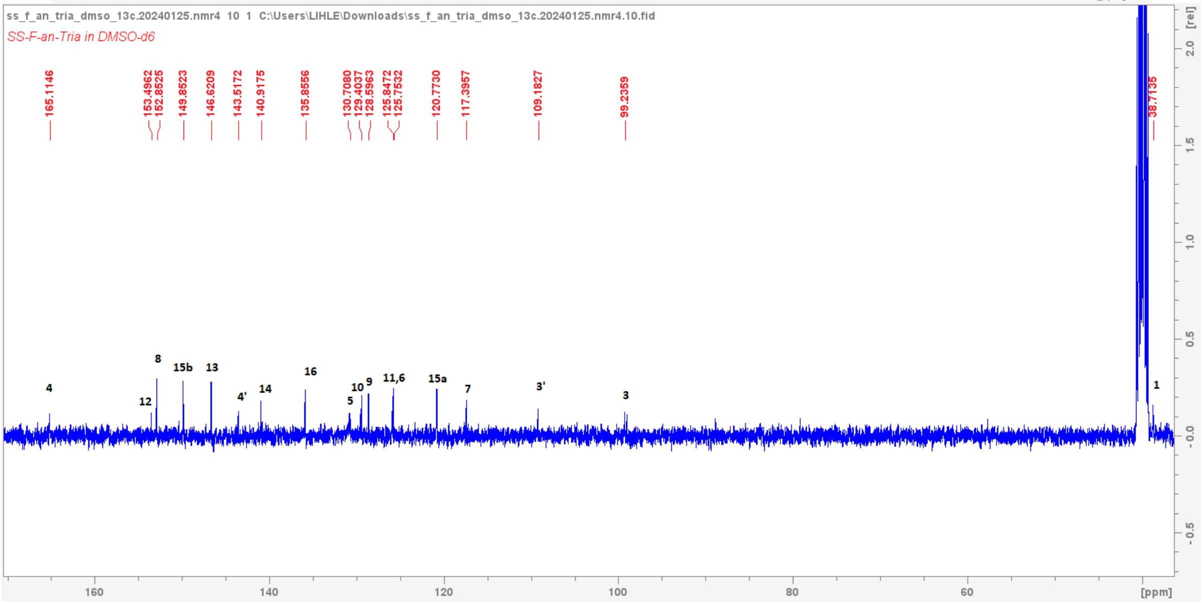

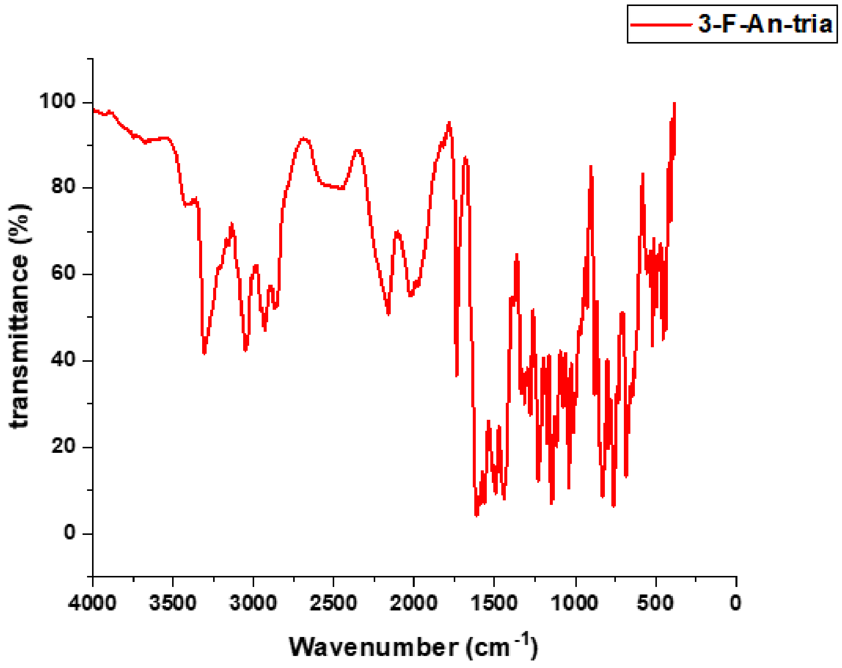

3-F-An-tria
transmittance (%)
Wavenumber (cm^{-1})

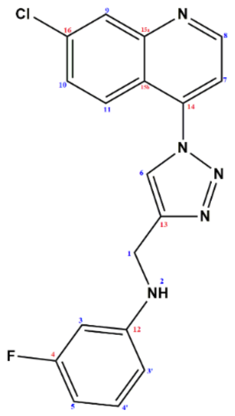

Cl
N
N
N
N
NH
F

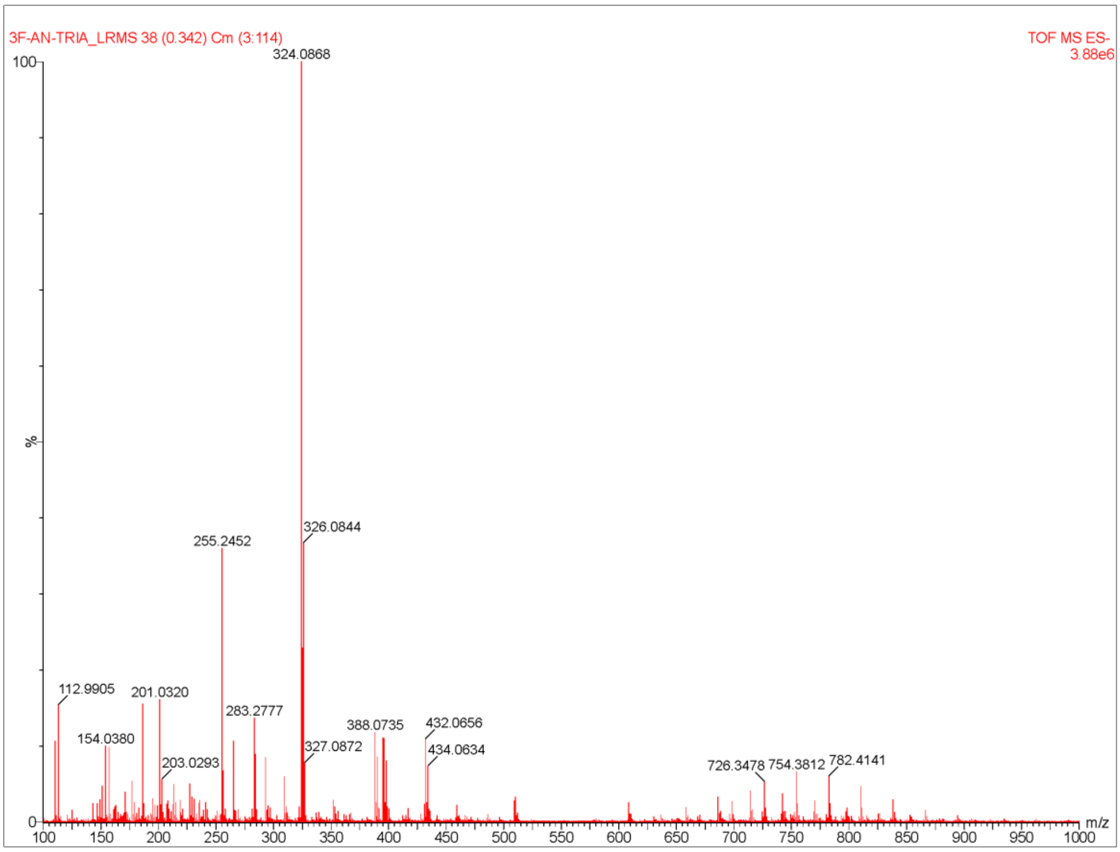

3F-AN-TRIA_LRMS 38 (0.342) Cm (3:114)
TOF MS ES-
3.88e6
324.0868
326.0844
255.2452
112.9905
201.0320
283.2777
154.0380
388.0735
432.0656
327.0872
434.0634
203.0293
726.3478
754.3812
782.4141
%
m/z

(1-(7-Chloro-4-qiunolinyl)-1*H*-1,2,3-triazole-4-methyl)-3-nitroaniline (11i):

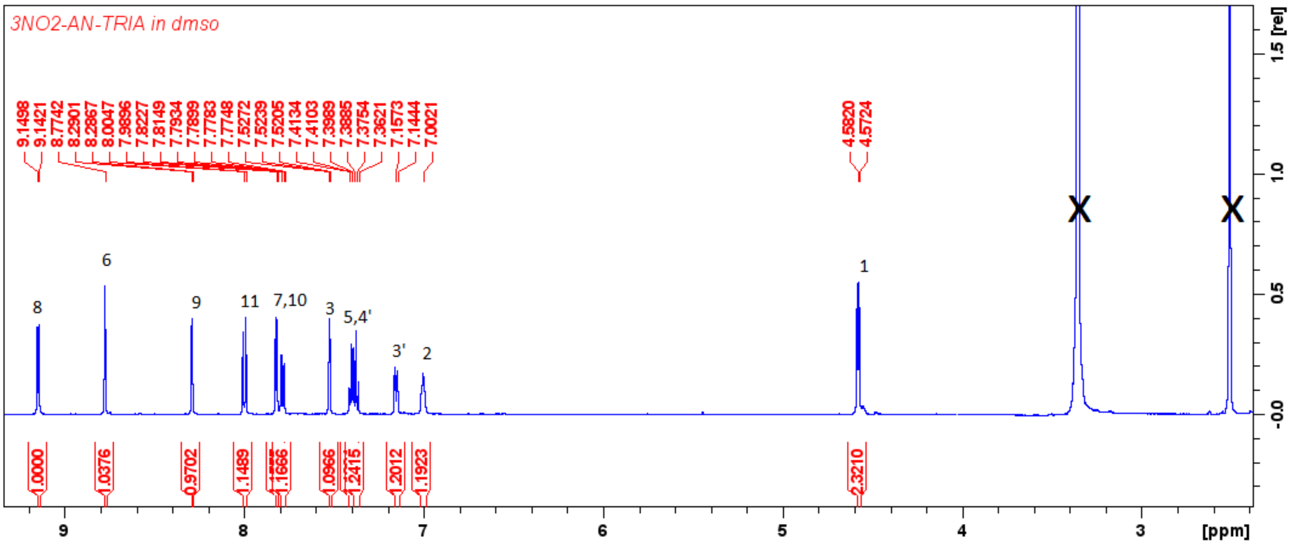

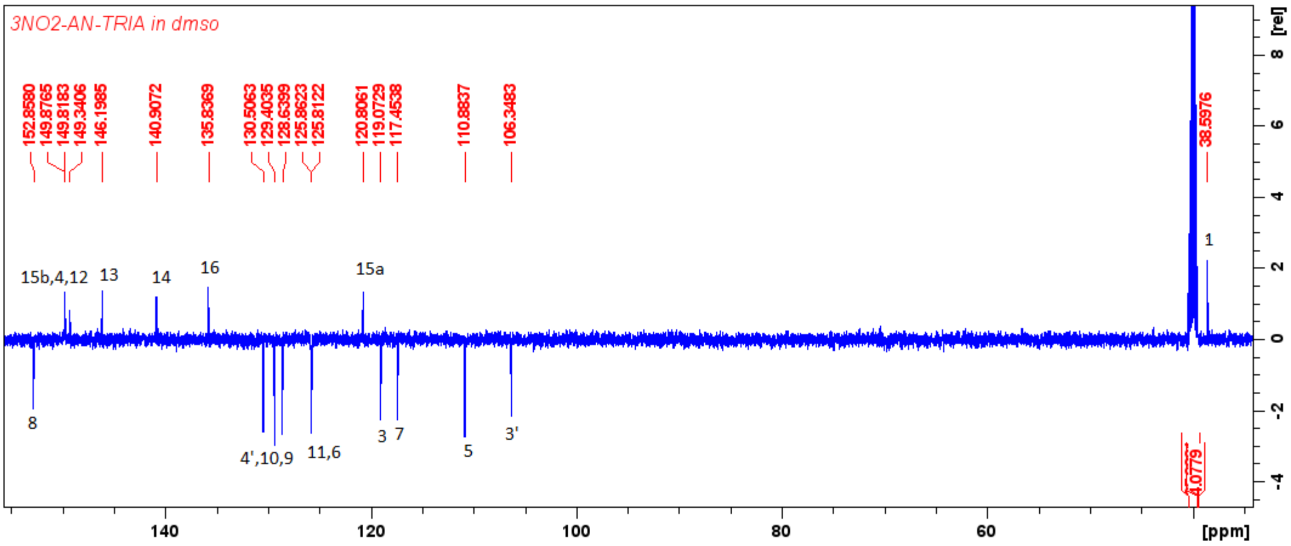

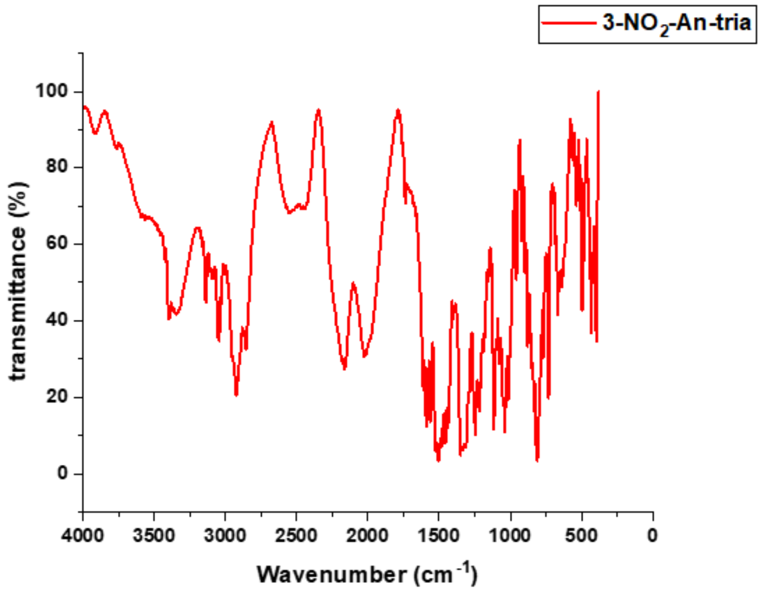
3-NO2-An-tria
transmittance (%)
Wavenumber (cm-1)
100
80
60
40
20
0
4000
3500
3000
2500
2000
1500
1000
500
0

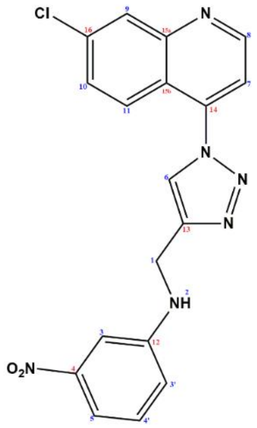
Cl
N
N
N
N
NH
O2N

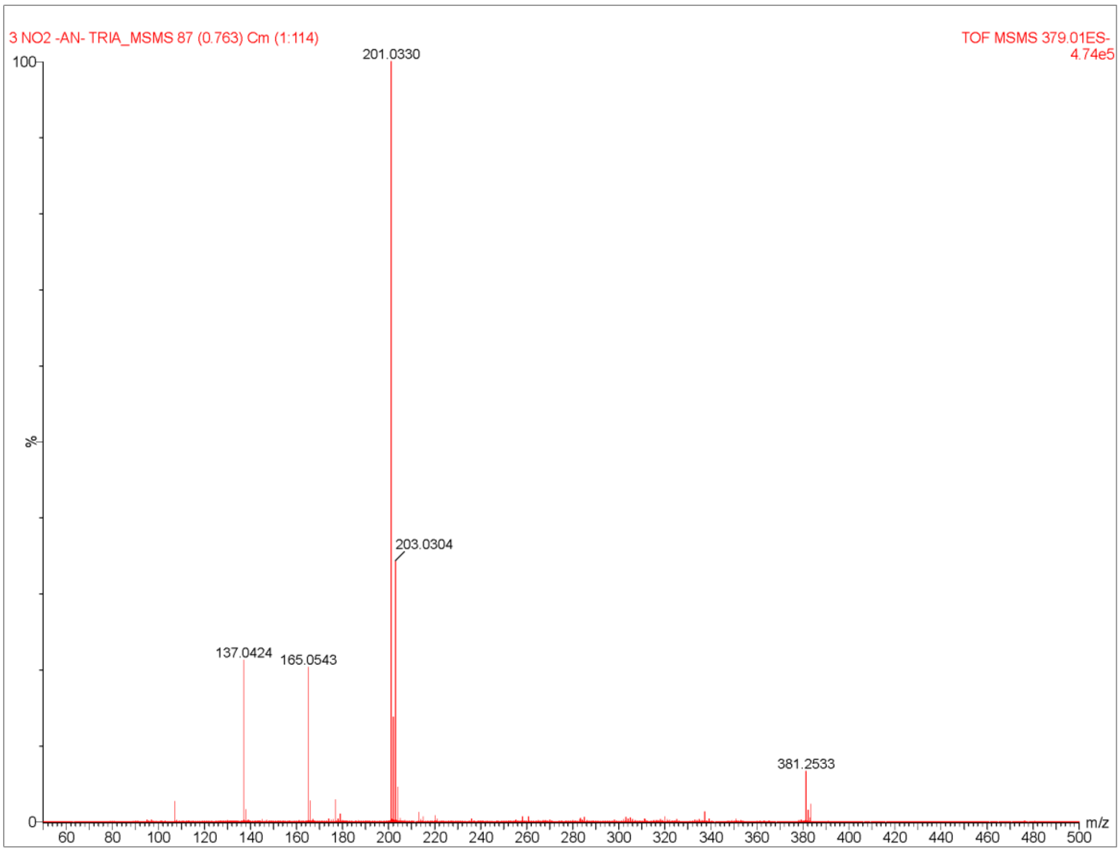
3 NO2 -AN- TRIA_MSMS 87 (0.763) Cm (1:114)
TOF MSMS 379.01ES-
4.74e5
100
%
0
201.0330
203.0304
137.0424
165.0543
381.2533
m/z
60
80
100
120
140
160
180
200
220
240
260
280
300
320
340
360
380
400
420
440
460
480
500

(1-(7-Chloro-4-quinolinyl)-1*H*-1,2,3-triazole-4-methyl)-4-methoxyaniline (11j):

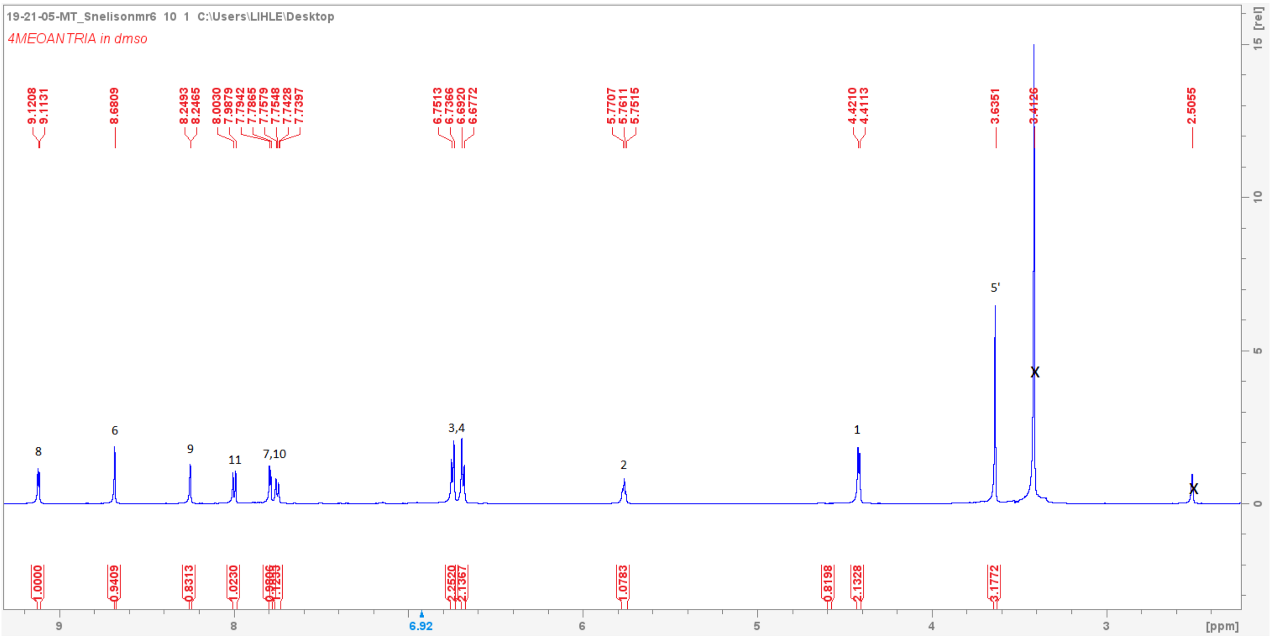

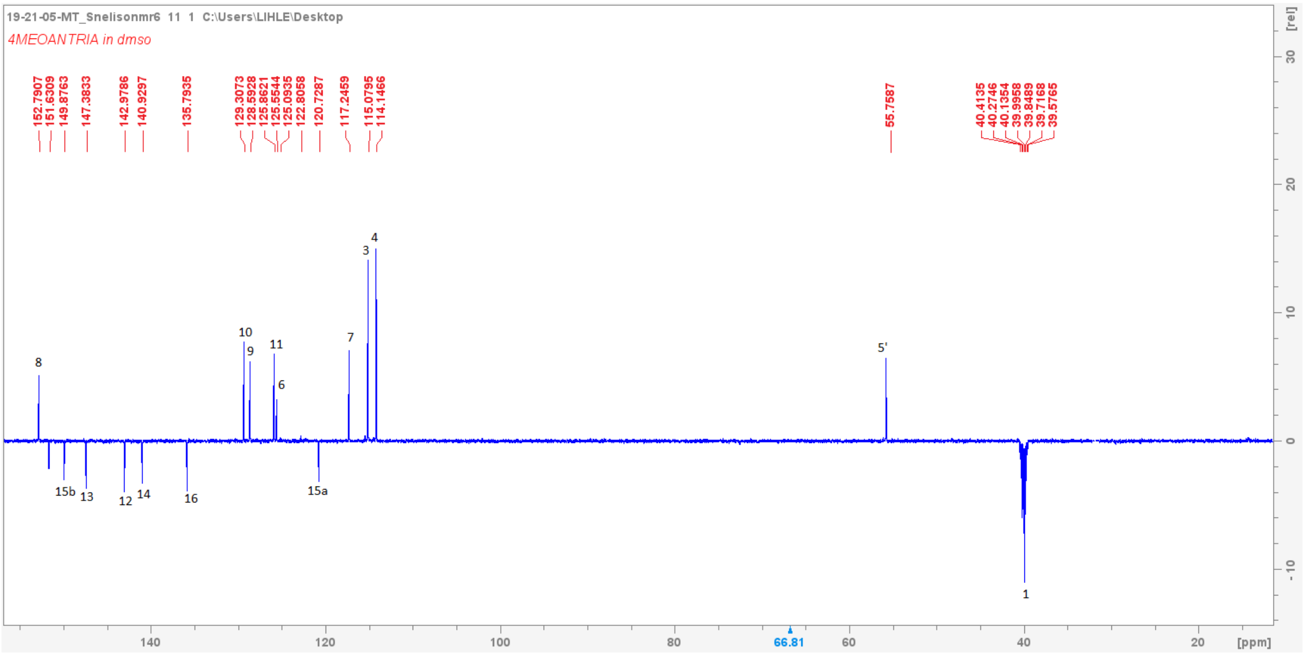

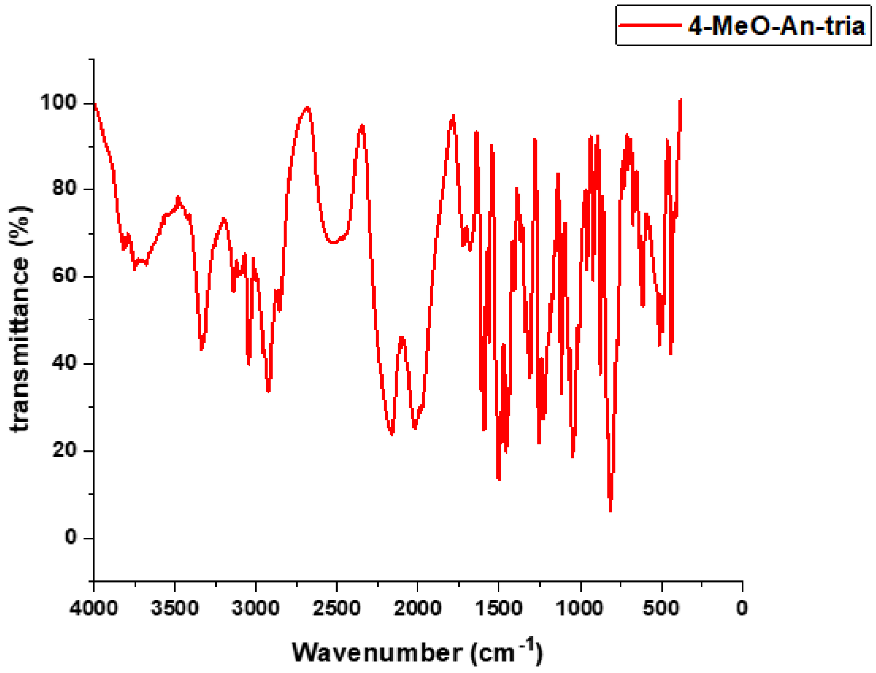
4-MeO-An-tria
transmittance (%)
100
80
60
40
20
0
4000
3500
3000
2500
2000
1500
1000
500
0
Wavenumber (cm^{-1})

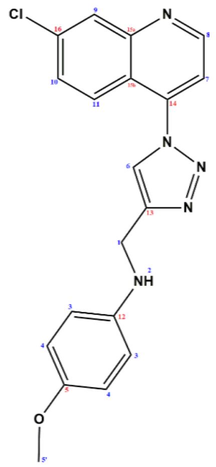
Cl
N
N
N
N
NH
O

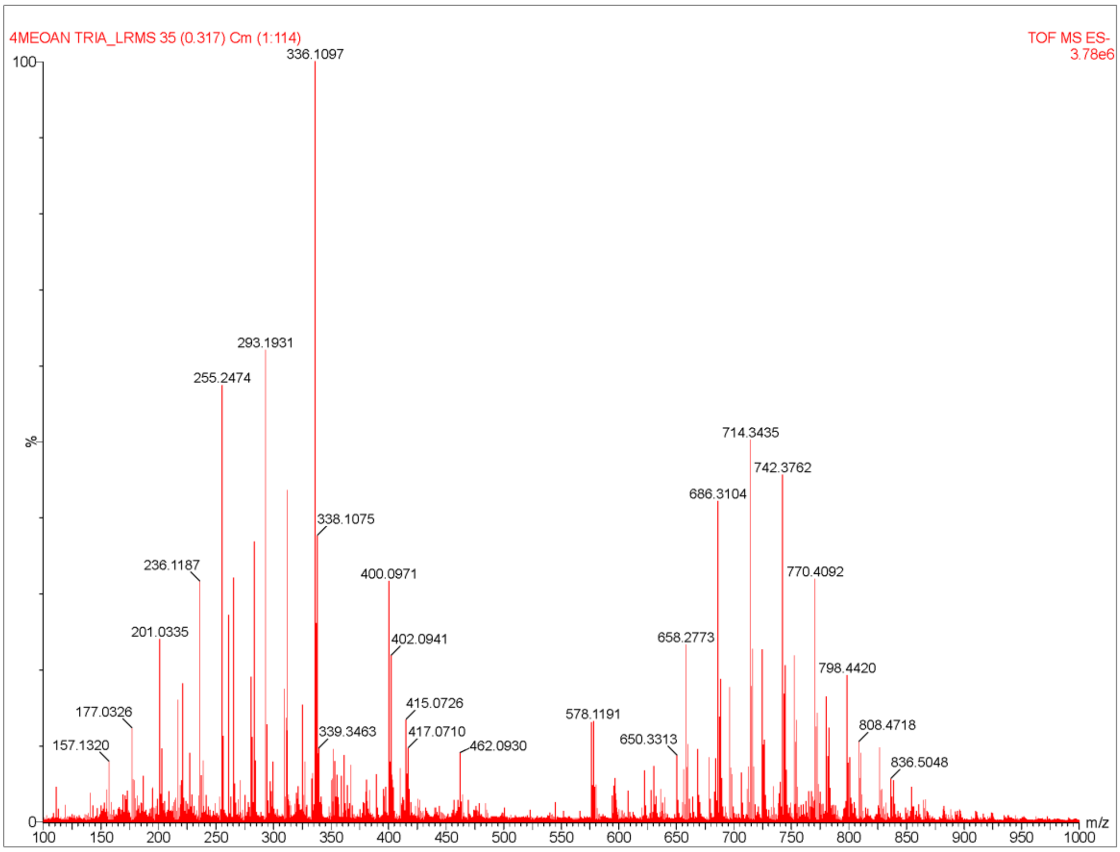
4MEOAN TRIA_LRMS 35 (0.317) Cm (1:114)
TOF MS ES-
3.78e6
100
%
0
336.1097
293.1931
255.2474
714.3435
742.3762
686.3104
338.1075
236.1187
400.0971
770.4092
201.0335
402.0941
658.2773
798.4420
177.0326
415.0726
578.1191
808.4718
157.1320
339.3463
417.0710
650.3313
462.0930
836.5048
m/z
100
150
200
250
300
350
400
450
500
550
600
650
700
750
800
850
900
950
1000

CYTOTOXICITY ASSAY DOSE-RESPONSE CURVES

11a

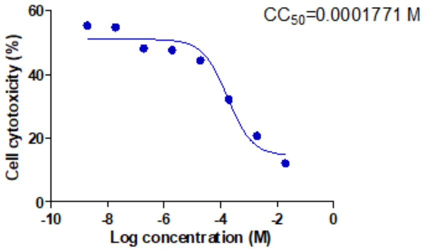

11b

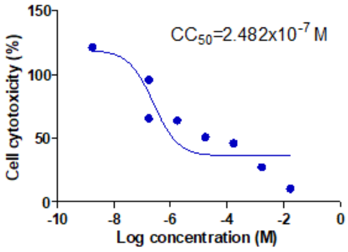

11c

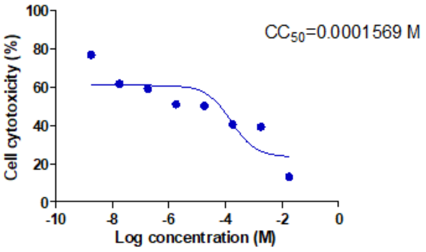

11d

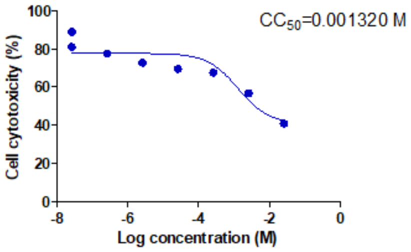

11e

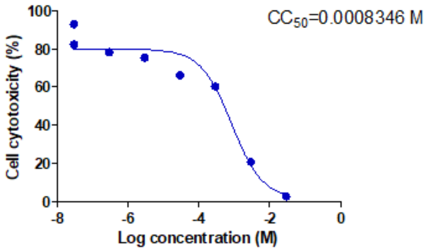

11f

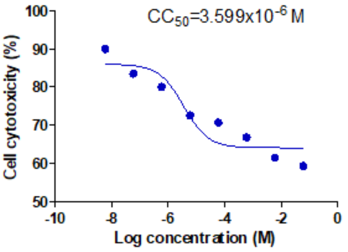

11g

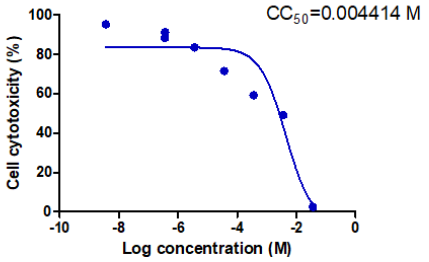

11h

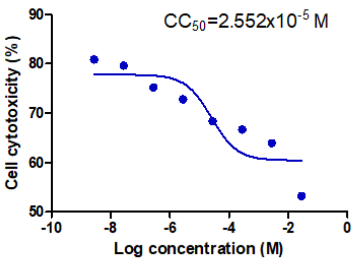

11i

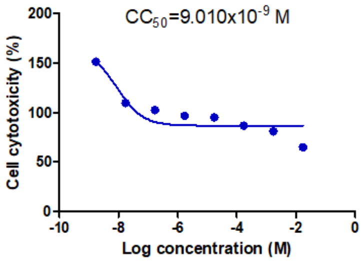

11j

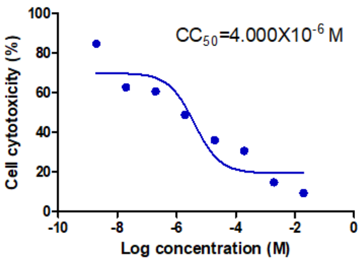

HIV-1 ASSAY DOSE-RESPONSE CURVES

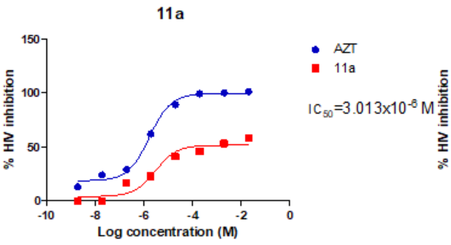

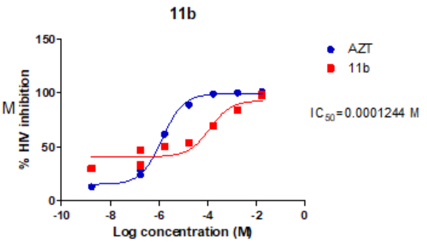

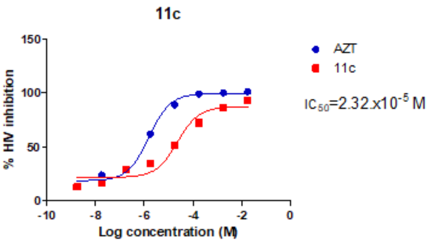

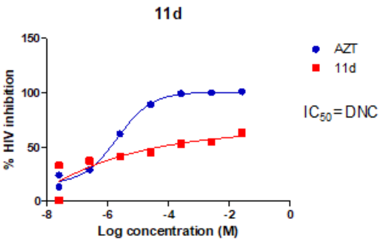

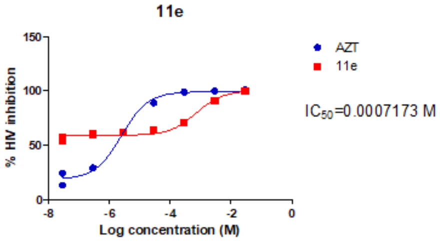

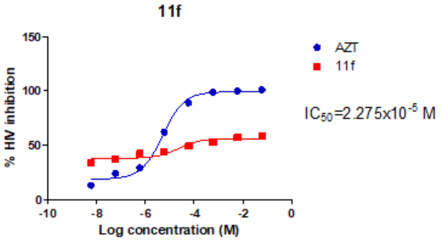

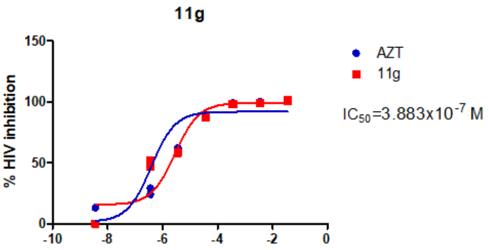

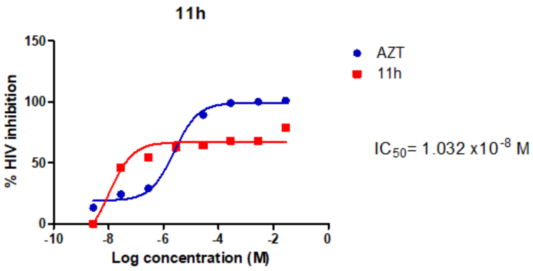

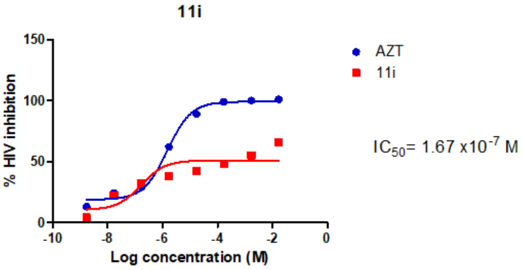

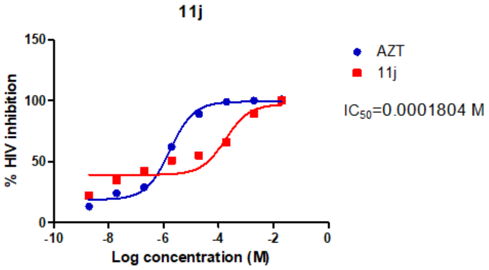

Supplement: Supplementary file 1 [file molecules-30-02119-s001.zip › molecules-3608139-supplementary.pdf]
